# tRNA modification reprogramming contributes to artemisinin resistance in *Plasmodium falciparum*

**DOI:** 10.1038/s41564-024-01664-3

**Published:** 2024-04-17

**Authors:** Jennifer L. Small-Saunders, Ameya Sinha, Talia S. Bloxham, Laura M. Hagenah, Guangxin Sun, Peter R. Preiser, Peter C. Dedon, David A. Fidock

**Affiliations:** 1 Division of Infectious Diseases, Department of Medicine, Columbia University Irving Medical Center, New York, NY USA; 2 Center for Malaria Therapeutics and Antimicrobial Resistance, Columbia University Irving Medical Center, New York, NY USA; 3 School of Biological Sciences, Nanyang Technological University, Singapore, Singapore; 4 Antimicrobial Resistance IRG, Singapore MIT Alliance for Research and Technology, Singapore, Singapore; 5 Department of Microbiology and Immunology, Columbia University Irving Medical Center, New York, NY USA; 6 Department of Biological Engineering, Massachusetts Institute of Technology, Cambridge, MA USA

**Keywords:** Parasite biology, Parasite genetics

## Abstract

*Plasmodium falciparum* artemisinin (ART) resistance is driven by mutations in kelch-like protein 13 (PfK13). Quiescence, a key aspect of resistance, may also be regulated by a yet unidentified epigenetic pathway. Transfer RNA modification reprogramming and codon bias translation is a conserved epitranscriptomic translational control mechanism that allows cells to rapidly respond to stress. We report a role for this mechanism in ART-resistant parasites by combining tRNA modification, proteomic and codon usage analyses in ring-stage ART-sensitive and ART-resistant parasites in response to drug. Post-drug, ART-resistant parasites differentially hypomodify mcm^5^s^2^U on tRNA and possess a subset of proteins, including PfK13, that are regulated by Lys codon-biased translation. Conditional knockdown of the terminal s^2^U thiouridylase, PfMnmA, in an ART-sensitive parasite background led to increased ART survival, suggesting that hypomodification can alter the parasite ART response. This study describes an epitranscriptomic pathway via tRNA s^2^U reprogramming that ART-resistant parasites may employ to survive ART-induced stress.

## Abstract (web summary)

Artemisinin-resistant *Plasmodium falciparum* can use tRNA modification reprogramming and codon bias translation as an epitranscriptomic response to survive artemisinin-induced stress.

## Main

Malaria, caused by *Plasmodium* parasites, resulted in 249 million cases and 608,000 deaths in 2022 (ref. ^1^). *Plasmodium falciparum*, the deadliest species, has a 48 h asexual blood stage (ABS) in human red blood cells (RBCs). Artemisinin (ART)-based combination therapies are first-line treatments for uncomplicated malaria and pair a short-acting yet highly potent ART derivative with a longer-acting partner drug^2^. ART partial resistance is widespread across Southeast Asia and has now been detected in Africa^3,4^. Point mutations in *P.* *falciparum* *k13*, including C580Y and R539T, are the major drivers of ART resistance^5–12^, associating with delayed parasite clearance in patients and in vitro resistance as defined using a ‘ring-stage survival assay’ (RSA)^13^.

ART, upon activation by haem, kills parasites by alkylating biomolecules and exerting general proteotoxic and oxidative stress, leading to widespread cellular damage^14^. The mechanisms behind mutant K13-mediated early ring-stage resistance remain partially understood, although reduced haemoglobin endocytosis plays a central role^15,16^. Mutant K13 has also been associated with upregulation of the unfolded protein response and the ubiquitin–proteasome system and enhancement of stress responses^15–24^. This evidence explains many aspects of ring-stage survival, but not how or why a subset of parasites enters quiescence and reinitiates development upon drug removal, a mechanism highly suited for the short half-life of ART^15,16,25^. Epigenetic regulation may be involved^26–29^. After drug exposure, K13-mutant parasites lengthen the duration of their ring-stage development, alter their metabolism and initiate translational repression, while continuing to maintain functional mitochondria^20,25,30–33^. This phenotype is similar to that of antibiotic-resistant bacterial persister cells, whereby a subpopulation tolerate stress without genetic modifications^34,35^. Given that only a small, yet reproducible, subset of K13-mutant parasites survive drug exposure^7,15,21,36^, we hypothesized that they may exploit differential changes in epigenetic and epitranscriptomic stress response pathways to enable survival.

*P.* *falciparum* has exceptionally few transfer RNA isoacceptors (45) to translate proteins from the set of 61 codons^37^. These tRNAs have highly conserved chemical modifications^38–41^ that enable them to differentiate synonymous codons encoding the same amino acids. These modifications, mediated by specific tRNA methyltransferases, can occur on the anti-codon loop or the tRNA body^42,43^. Modifications, especially those at the wobble position 34, can alter the rate and fidelity of translation^44,45^. One such modification, mcm^5^s^2^U, is necessary for improved translation of Lys, Glu and Gln codons ending in A by allowing for Watson–Crick and non-Watson–Crick base pairing at the third position of the anti-codon^40,45–48^. Altered tRNA modifications serve critical roles in responses to stress, tRNA stability, control of cell growth and disease pathogenesis^49–54^. Organisms can expand or limit their tRNA decoding capability, leading to decoding of cognate codons that are over- or under-represented in messenger RNAs^55–58^. This leads to enhanced translational elongation and selective ‘just in time’ up- or downregulation of codon-biased proteins^45,50,59^.

tRNA modifications have been characterized in model organisms^60,61^. In *P.* *falciparum*, tRNA modification reprogramming fine-tunes stage-specific protein expression by enhancing translation efficiency of select codon-biased transcripts^62^. Interestingly, transcriptomic analysis of mutant K13 parasites revealed a significant increase in U_34_ tRNA modifying enzymes after ART exposure^23^, suggesting that reprogramming these modifications may be an epitranscriptomic mechanism used by resistant parasites to adapt to ART-induced stress. Despite the importance of tRNA modification reprogramming in bacterial and yeast stress responses, cancer and human diseases^60,61,63^, the role of tRNA modifications in *P.* *falciparum* drug resistance or stress responses has yet to be explored. In this Article, we use mass spectrometry-based tRNA modification analysis and proteomics on isogenic K13-mutant parasites combined with studies of a conditionally regulated tRNA thiouridylase, PfMnmA, to demonstrate that tRNA modification reprogramming plays a previously unrecognized role in the ART stress response.

## Results

### tRNA modification reprogramming occurs in ART-R parasites post-drug

**Fig. 1:**
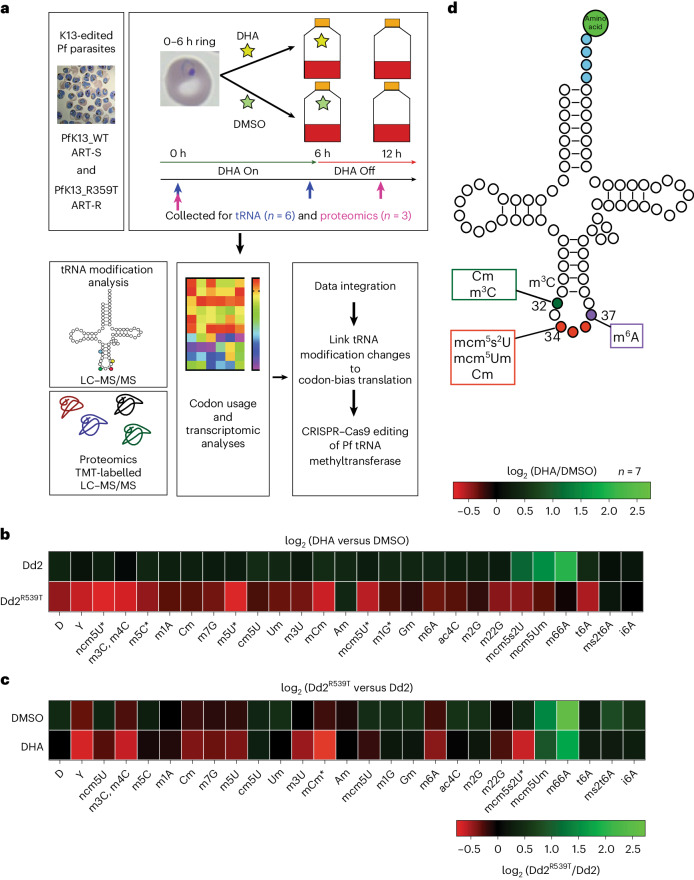
ART-R parasites differentially alter their tRNA modifications in response to ART stress. **a**, The workflow for data generation and integration to assess tRNA modification and proteomic changes as well as codon bias translation. Isogenic, edited Dd2 (ART-S harbouring K13-silent binding-site mutations) and Dd2^R539T^ (ART-R K13 R539T mutant) *P. falciparum* (Pf) parasites^7^ were sorbitol synchronized to early ring stages (0–6 hpi) then pulsed with either 700 nM DHA or 0.1% DMSO. For tRNA experiments, samples were collected at 0 and 6 h post-exposure. For proteomics, samples were collected at 0 h and 12 h, with the drug having been removed by wash offs at 6 h. tRNA molecules were purified and modifications analysed by LC–MS/MS. Proteomics was performed using TMT-tagged samples and LC–MS/MS (Methods). Codon bias analysis was run using a codon-counting algorithm and further analysed by principal component analysis. These data were combined to identify particular modification changes that led to codon bias changes. Findings were validated using a cKD of the tRNA 2-thiouridylase PfMnmA. **b**,**c**, Changes in the relative quantities of modified ribonucleosides, as quantified by LC–MS/MS in total tRNA extracted from parasites at the timepoints indicated in **a**. Average fold-change values (range −0.8 to 2.7) were calculated for DHA treatment versus DMSO treatment of the Dd2^R539T^ or Dd2 parasites (relative to *t* = 0 values) (**b**) or Dd2^R539T^ parasites versus Dd2 parasites for either DMSO treatment or DHA treatment (**c**). The results were subjected to hierarchical clustering analysis (log_2_ transformed data). *n* = 7 independent biological replicates. Statistics were performed using two-tailed *t*-tests on data normalized to *t* = 0, *P < 0.05% (Source data). D, dihydrouridine; Y, pseudouridine. **d**, A schematic of the tRNA secondary structure with location of key modifications. Wobble positions 34–36 are shown in red, position 37 is shown in purple and position 32 is shown in green. Source data

**Extended Data Fig. 1:**
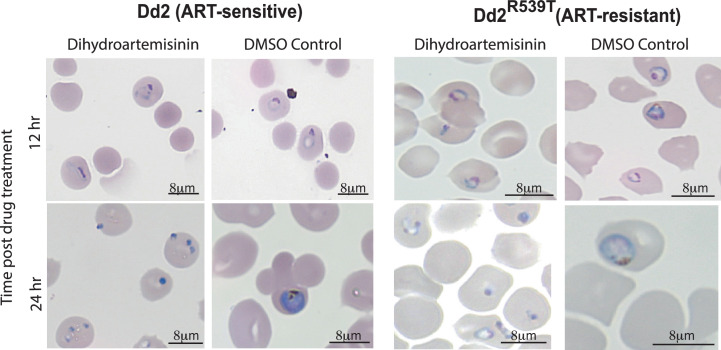
A population of Dd2^R539T^ parasites, but not Dd2 parasites, persist as ring stages after dihydroartemisinin (DHA) treatment. Giemsa-stained parasite images illustrate pyknotic parasites in DHA-pulsed Dd2 parasites but a subset of surviving ring-stage parasites in DHA-pulsed Dd2^R539T^, as determined 12 h and 24 h post drug treatment. DMSO-treated parasites in both lines progressed normally through the ABS cycle. ~25% of Dd2^R539T^ parasites survived. At least 500 cells were counted for each condition.

To assess whether ART-resistant (ART-R) parasites differentially alter their tRNA modification profiles as compared with ART-sensitive (ART-S) lines in response to a pulse of dihydroartemisinin (DHA), we used a modified, large-scale RSA^13^, which measures the survival of newly invaded intra-erythrocytic ring-stage parasites exposed to a brief, 6 h pulse of the ART active metabolite, DHA. This assay was combined with previously described workflows to quantify tRNA modifications and link them to proteomic changes and codon use bias (Fig. 1a)^62^. We selected the Asian, ART-S Dd2 parasite (expressing the wild-type (WT) *k13* allele with silent binding-site mutations) and its isogenic, ART-R Dd2^R539T^ line that expresses the K13 R539T variant^7,12,23^. Initial RSAs confirmed the ART resistance phenotype in Dd2^R539T^ parasites, with a survival level of 25% at 24 h post-DHA treatment, as compared with <1% survival in Dd2 parasites, consistent with earlier reports^7,23^ (Extended Data Fig. 1). Dd2^R539T^ parasites that survived DHA treatment remained as ring stages after 24 h.

To examine tRNA modifications, tRNA from highly synchronized early ring-stage (0–6 h post invasion (hpi)) parasites was prepared and purified at *t* = 0 and after a 6 h pulse of 700 nM DHA or dimethylsulfoxide (DMSO) vehicle control (Fig. 1a). tRNA modifications were analysed using liquid chromatography-coupled mass spectrometry (LC–MS/MS)^62^. Lines were cultured simultaneously for each biological replicate to minimize variation in temperature, nutrient supply and other stressors^41^.

We detected 27 tRNA modifications with high confidence, similar to the 28 detected in earlier *P.* *falciparum* profiling^62^. To standardize analyses, all modification levels were normalized to *t* = 0 for each line and biological replicate. First, we examined whether tRNA modifications differed between DHA or DMSO treatments, for both Dd2 and Dd2^R539T^ parasites (Fig. 1b). ART-S Dd2 parasites had minimal changes in their tRNA modification levels after DHA exposure relative to DMSO, with only mcm^5^s^2^U, mcm^5^Um and m^6,6^A showing evidence of a slight increase after DHA treatment compared with DMSO. In marked contrast, Dd2^R539T^ parasites had a global decrease in tRNA modifications after DHA treatment relative to DMSO. Significant decreases (*P* < 0.05, two-tailed Student’s *t*-test) were observed with ncm^5^U, m^5^C, m^5^U, mcm^5^U and m^1^G. Two other modifications, mcm^5^s^2^U and mcm^5^Um, also decreased in DHA-treated Dd2^R539T^ and, notably, increased in DHA-treated Dd2 parasites. This finding suggested differential tRNA reprogramming between the K13 mutant and WT parasites in response to DHA.

To identify changes specific to ART-R parasites after DHA exposure, we compared tRNA modification levels in Dd2^R539T^ versus Dd2 parasites by comparing the ratio of tRNA modifications for Dd2^R539T^ versus Dd2 for each of DMSO and DHA treatments (Fig. 1c). These two lines showed minimal differences after DMSO exposure, with none reaching significance. Nonetheless, two modifications (mcm^5^Um and m^6,6^A) increased in DMSO-treated parasites, although these were also observed in DHA-treated parasites, suggesting a drug-independent effect that probably reflects temporal changes. DHA treatment resulted in 12 other modifications that were decreased in Dd2^R539T^ parasites, to an extent greater than observed post-DMSO exposure. Two of these modifications, mcm^5^s^2^U and mCm, attained significance (*P* < 0.05, two-tailed Student’s *t*-test), suggesting that their targeted reprogramming may be a specific response to DHA treatment in mutant K13 parasites (Fig. 1c). The mcm^5^s^2^U modification combines a 5-carboxymethonylmethyl (mcm^5^) group and a 2-thio (s^2^) group on the U_34_ position, with each part of the modification having its own biosynthetic pathway^47^.

The mcm^5^s^2^U modification was of particular interest as (1) it is located on the wobble position 34 of the Lys, Glu and Gln tRNAs (Fig. 1d) and therefore has the potential to alter translation and (2) an earlier study noted that genes involved in 2-thio (s^2^) biosynthesis were differentially expressed in K13 mutant versus WT isogenic parasites following DHA exposure^23^. These data suggest that ART-R parasites differentially alter their tRNA modification profile in response to ART stress, raising the possibility that these changes may have a direct link to translation of proteins important for the stress response and/or emergence from quiescence.

### ART-R parasites alter their proteome after DHA exposure

**Fig. 2:**
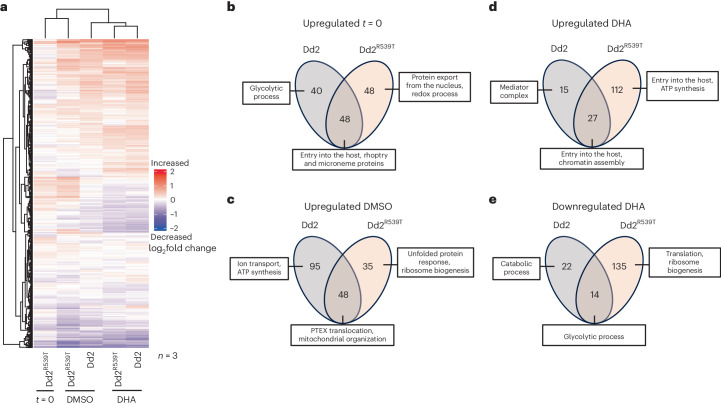
The Dd2^R539T^ parasite proteome is differentially altered after DHA exposure. TMT-tagged proteomics analysis identified 1,315 proteins with 40,955 PSMs from Dd2 or Dd2^R539T^ parasites at 0 h or 12 h after a 6 h DHA or DMSO pulse. Isogenic, edited Dd2 and Dd2^R539T^ parasites^7^ were highly sorbitol synchronized to early ring stages (0–6 hpi) then pulsed with either 700 nM DHA or 0.1% DMSO. Samples were collected at 0 h and 12 h, with the drug having been removed by wash offs at 6 h (Fig. 1a). **a**, A heat map of hierarchical clustering analysis of log_2_-transformed fold changes in the protein levels of each proteome normalized to the Dd2 *t* = 0 proteome. **b**–**e**, Venn diagrams showing unique and common significant proteins and their GO terms in the Dd2 or Dd2^R539T^ parasite proteomes that were upregulated at 0 h (**b**), upregulated post-DMSO vehicle control (**c**) and upregulated (**d**) or downregulated post DHA (**e**). PTEX, Plasmodium translocon of exported proteins. Source data

**Extended Data Fig. 2:**
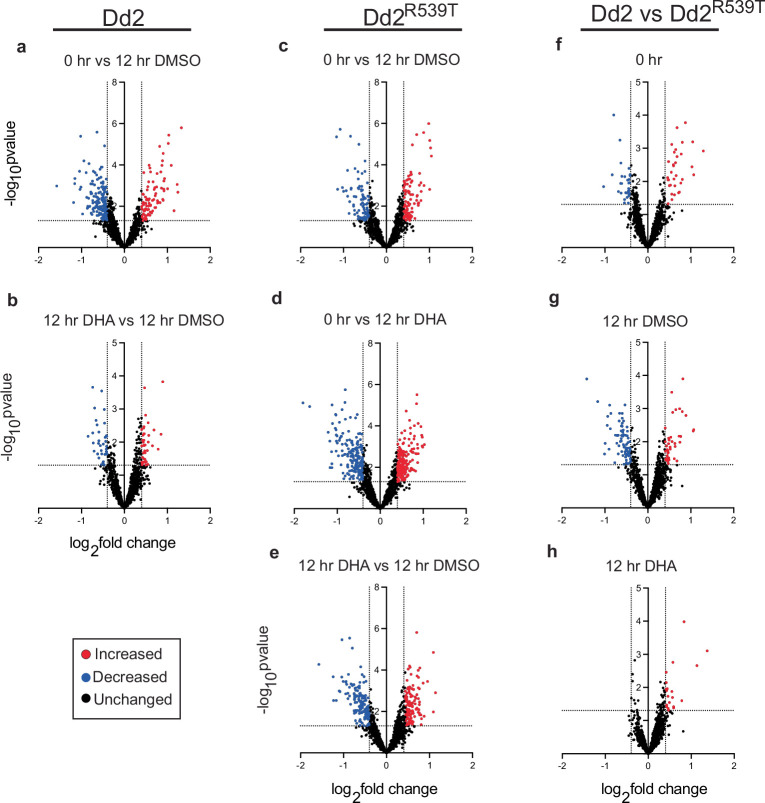
Volcano plots of proteomic data comparing the Dd2 and Dd2^R539T^ parasite lines at 0 h and post DHA or DMSO. TMT-tagged proteomics analysis resulted in 1,315 proteins with 40,955 peptide spectral matches from Dd2 or Dd2^R539T^ parasites at 0 h or 12 h (6 h DHA or DMSO pulse and recovery). Isogenic, edited Dd2 (binding-site mutant, artemisinin-sensitive) and Dd2^R539T^ (artemisinin-resistant) parasites^11^ were highly sorbitol synchronized to early ring stages (0-6 h post invasion, hpi) then pulsed with either 700 nM DHA or 0.1% DMSO. Samples were collected at t=0 (pink arrows, Fig. 1a), parasites underwent thorough drug wash-off at 6 h, and samples were collected at 12 h (pink arrows, Fig. 1a). Volcano plots showing p-values (-log_10_) versus log_2_ transformed fold changes for differentially changing proteins in Dd2 parasite proteomes (**first column**) for (**a**) 0 h vs 12 h DMSO and (**b**) 12 h DHA vs 12 h DMSO; Dd2^R539T^ parasite proteomes (**second column**) for (**c**) 0 h vs 12 h DMSO, (**d**) 0 h DHA vs 12 hr DHA and (**e**) 12 h DHA vs 12 h DMSO; and Dd2 vs. Dd2^R539T^ parasite proteomes (**third column**) at (**f**) 0 h or after (**g**) DMSO or (**h**) DHA treatment. Red circles: increased proteins with significant changes between the two conditions; blue: decreased proteins with significant changes between the two conditions; black: unaltered proteins between the two conditions. Significant changes were assessed as log_2_ fold change > 0.4 or < −0.4 and adjusted p value < 0.05. p-value calculations were performed using a linear mixed-effects model fit performed in the MSstatsTMT script^96^.

We next evaluated changes in the Dd2 and Dd2^R539T^ proteomes after DHA or DMSO exposure. Samples were collected from synchronized ring-stage parasites (0–6 hpi) at *t* = 0 (Fig. 1a). These parasites were exposed to either 700 nM DHA or DMSO vehicle control for 6 h, washed and allowed to recover in drug-free media until 12 h post-pulse, when they were collected for proteomic analyses (Extended Data Fig. 1). We identified a total of 1,315 proteins based on 40,955 peptide spectral matches (PSMs) across all samples, using quantitative isobaric tags (tandem mass tag (TMT)) with a labelling efficiency >99%. We represented these proteins as a heat map that depicts relative changes at *t* = 12 for both DHA and DMSO samples compared with the Dd2 *t* = 0 proteome. Unsupervised data clustering found that in the 12 h samples, compared with the Dd2 *t* = 0 samples, DHA-treated Dd2^R539T^ and Dd2 parasites showed very similar proteome profiles. In contrast, substantial differences were observed between Dd2 and Dd2^R539T^ in the DMSO controls (Fig. 2a). We then compared our different experimental conditions to ascertain similarities and differences between each proteome (Extended Data Fig. 2).

We compared differentially regulated proteins at *t* = 0 versus 12 h post-DMSO for Dd2 and Dd2^R539T^ parasites. For the *t* = 0 Dd2 sample, 25 of the 88 proteins enriched compared with the 12 h timepoint had a Gene Ontology (GO) enrichment category of host cell entry (Fig. 2b, Supplementary Table 1 and Supplementary Results). Proteins involved in response to unfolded proteins were significantly upregulated in Dd2^R539T^ parasites at 12 h post-DMSO, but not in Dd2 parasites (Fig. 2c and Supplementary Table 1). We also compared differentially regulated proteins in Dd2 versus Dd2^R539T^ parasites after ART versus DMSO exposure. Dd2^R539T^ parasites showed a strong downregulation in genes involved in translation (Fig. 2d,e, Supplementary Table 1 and Supplementary Results)

To identify the selective response of DHA-treated mutant parasites, we examined proteins differentially regulated in DHA-treated Dd2^R539T^ parasites that did not significantly change in DHA-treated Dd2 parasites. Forty-four proteins were significantly upregulated and were involved in protein refolding and mitochondrial physiology. Of the 70 downregulated proteins, several were involved in translation, with 14 proteins involved in ribosome biogenesis (Supplementary Table 1).

### The ART-R parasite proteome displays codon use bias

**Fig. 3:**
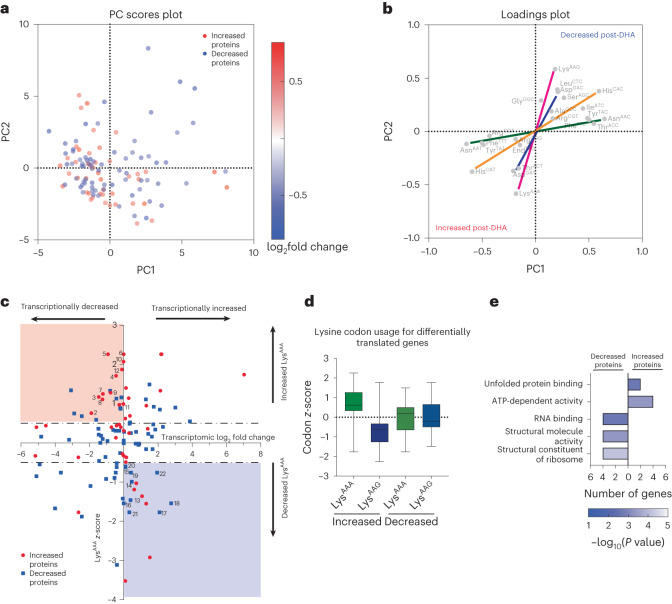
A subset of proteins, including K13, are regulated by lysine codon bias translation in Dd2^R539T^ parasites. **a**, The top 44 upregulated proteins and bottom 70 downregulated proteins in Dd2^R539T^ parasites after DHA exposure were analysed for codon usage patterns (Source data). The codon usage percentages in each gene were used to prepare a data matrix for principal component analysis. The scores plot shows codon use distinction between increased proteins and decreased proteins, with changes greatest in decreased proteins along PC1. **b**, The corresponding loadings plot for **a** shows codons contributing most strongly to this separation. For ease of visualization, unchanged codons were removed with the full loadings plot shown in Extended Data Fig. 3a. Cognate codon pairs significantly contributing to this separation are joined by coloured lines (Lys, pink; Asp, blue; His, orange and Asn, green). **c**, An assessment of differentially regulated proteins for lysine codon usage versus transcriptional direction post-DHA in Dd2^R539T^ parasites. Increased proteins and decreased proteins were evaluated for Lys^AAA^ codon usage with *z*-scores >0.5 or <−0.5 considered significant (*y* axis). Transcriptomic data from Mok et al.^23^ were analysed for Dd2^R539T^ parasites after a 6 h DHA pulse and assessed for log_2_ fold change compared with parasites at timepoint 0 (Source data and Extended Data Fig. 3). Candidate proteins regulated by Lys codon bias translation were considered those that displayed Lys codon bias and had either increased abundance with decreased translation (red-shaded region, Supplementary Table 3) or decreased abundance with increased translation (blue-shaded region, Supplementary Table 4). Proteins that met criteria are numbered and detailed in Table 1. **d**, Box-and-whisker plot showing Lys codon usage for all differentially translated proteins. The *z*-score for Lys^AAA^ codon usage for increased proteins and decreased proteins as compared with the *z*-score for Lys^AAG^ codon usage for increased and decreased proteins. Data were derived from *n* = 3 independent biological replicates. Centre line, median; box limits, upper and lower quartiles; and whiskers, minimum and maximum values. **e**, GO analysis for increased and decreased codon bias proteins with the number of genes per GO slim term on the *x* axis. The heat map shading represents −log_10_ *P* values (two-tailed Fisher exact test) (Supplementary Tables 3 and 4). Source data

**Extended Data Fig. 3:**
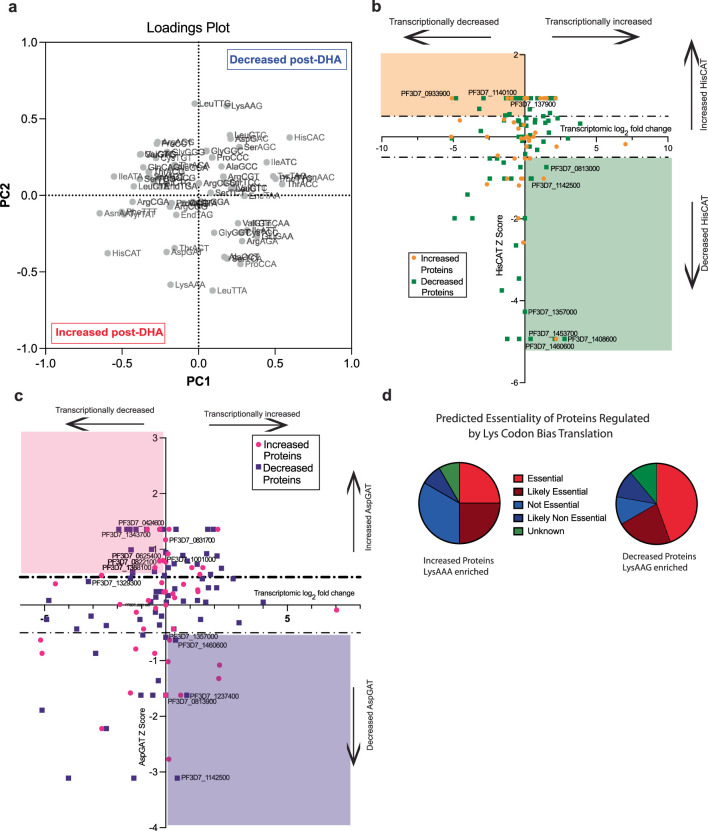
A subset of proteins are regulated by histidine and aspartate codon bias translation in Dd2^R539T^ parasites. (**a**) Full loadings plot as shown in Fig. 3b without codons removed. Assessment of proteins with differential abundance and their His (**b**) or Asp (**c**) codon usage vs transcriptional direction post-DHA in Dd2^R539T^ parasites. Increased proteins (His-orange circles; Asp- circles) and decreased proteins (His- green squares; Asp- purple squares) were evaluated for His^CAT^ (**b**) or Asp^GAT^ (**c**) codon usage with *z*-scores > 0.5 or < −0.5 considered significant (y-axis). Transcriptomic data from Mok et al.^22^ were analyzed for Dd2^R539T^ parasites after a 6 h DHA pulse and assessed for log_2_ fold change compared to time point t=0 (Source Data for both Fig. 3 and Extended Data Fig. 3). Candidate proteins for those regulated by His or Asp codon bias translation were considered proteins that displayed codon-bias and had either increased abundance with decreased transcription (His (**b**)- orange shaded region, Supplementary Table 5; Asp (**c**)-pink shaded region, Supplementary Table 7) or decreased abundance with increased transcription (His (**b**)- green shaded region, Supplementary Table 6; Asp (**c**)-purple shaded region, Supplementary Table 8). (**d**) Predicted essentiality of lysine codon-bias translated proteins was determined by transposon mutagenesis^69^. Genes were classified as essential (light red), likely essential (dark red), not essential (light blue), likely not essential (dark blue) and unknown (green), with data noted in Supplementary Tables 3, 4. Source data

We tested whether biased use of synonymous codons occurred in the top up- or downregulated proteins identified in Dd2^R539T^ parasites after DHA exposure, as compared with Dd2^R539T^ parasites sampled at *t* = 0. We excluded proteins that were similarly up- or downregulated at the translational level in DMSO-treated Dd2^R539T^ and/or DHA-treated Dd2, to identify protein changes unique to DHA-treated Dd2^R539T^ parasites. To analyse these data, we employed a codon-counting algorithm to quantify codon usage patterns in the top 44 upregulated and bottom 70 downregulated proteins (that is, proteins >0.5 or <−0.5 log_2_ fold change in Dd2^R539T^ DHA versus *t* = 0 samples and between 0.5 and −0.5 log_2_ fold change for Dd2 DHA versus *t* = 0 samples). Principal component analysis revealed a separation in the codon usage patterns of these two groups, mainly in principal component 1 (PC1; Fig. 3a). The corresponding loadings plot demonstrated a strong association of three codons with the upregulated proteins Lys^AAA^, His^CAT^ and Asp^GAT^, with enrichment of their cognate codons (Lys^AAG^, His^CAC^ and Asp^GAC^) in the downregulated proteins (Fig. 3b). Lys^AAA/AAG^ was the greatest driver amongst codon pairs. Of note, the majority of codons were unchanged between up- and downregulated proteins (Extended Data Fig. 3a, major codon changes are shown in Fig. 3b).

We next searched for up- and downregulated proteins enriched for Lys^AAA^ or Lys^AAG^ as evaluated by *z*-scores ≥0.5. Among upregulated proteins, 48% were enriched for Lys^AAA^ compared with 16% for Lys^AAG^. In contrast, for downregulated proteins, 34% were enriched for Lys^AAG^ versus 23% for Lys^AAA^ (Supplementary Table 2). Differences in the usage of His and Asp cognate codons in the up- and downregulated proteins were less pronounced (Supplementary Table 2). Interestingly, the differentially regulated mcm^5^s^2^U modification (Fig. 1c,d) occurs on the U_34_ wobble position of Lys^AAA/AAG^ codons to regulate translational fidelity^44,47^, providing a mechanistic link between our tRNA reprogramming changes and the Lys codon bias translation noted above.

### Stress-response proteins show Lys codon-biased translation post-ART

To explore whether changes in protein levels were attributable to codon-biased translation rather than to transcriptional regulation, we searched for translationally up- or downregulated proteins that displayed Lys codon bias and whose transcript levels were unchanged or moving in opposite directions to their protein levels. We analysed published transcriptomic data^23^ that profiled highly synchronized Dd2^R539T^ isogenic parasites before and after a 6 h DHA pulse. Given the lag between transcript and protein level changes, we focused on altered protein levels 6 h after completing the DHA pulse in Dd2^R539T^ parasites. Dd2 parasites were not explored as they are effectively dead after 6 h of DHA exposure. In the Dd2^R539T^ line, 50% of upregulated proteins and 43% of downregulated proteins were found to change transcriptionally in the equal or opposite direction to the protein changes (Supplementary Table 2). By integrating the proteomic and transcriptomic changes and Lys^AAA^ codon usage (Fig. 3c), we identified a subset of 12 translationally upregulated proteins that were enriched for Lys^AAA^ and transcriptionally downregulated (Fig. 3c, Table 1 and Supplementary Table 3). We also identified a separate set of ten translationally downregulated proteins that were enriched for Lys^AAG^ but increased transcriptionally (Fig. 3c, Table 1 and Supplementary Table 4). Within this set of 22 differentially translated proteins in our DHA-treated ART-R parasites, the upregulated proteins showed a clear Lys codon bias (Fig. 3d). Importantly, not all up- or downregulated proteins displayed codon bias, nor did they all have opposing transcription profiles, suggesting that we had identified a unique subset of proteins regulated by Lys codon bias translation in the ART-R parasites. We performed a similar analysis for His and Asp codon pairs (Extended Data Fig. 3b,c) and identified similar, although smaller, sets of codon-biased regulated proteins (Supplementary Tables 5–8).

**Table 1:** Up- and downregulated lysine codon bias proteins

Increased proteins								Decreased proteins							
No.	Gene ID	Gene name	log_2_ protein fold change	Lys^AAA^ *z*-score	log_2_ RNA fold change	Other codon bias	Essential	No.	Gene ID	Gene name	log_2_ protein fold change	Lys^AAA^ *z-*score	log_2_ RNA fold change	Other codon bias	Essential
1	PF3D7_0625400	Uncharacterized protein	0.63	0.97	−0.26	Asp^GAT^	Yes	13	PF3D7_1460600	Inner membrane complex subcompartment protein 3	−0.50	−1.46	0.40	His^CAC^, Asp^GAC^	Unknown
2	PF3D7_0321100	Uncharacterized protein	0.59	0.76	−1.89	No	No	14	PF3D7_1142500	60S ribosomal protein L28	−0.51	−0.99	0.47	His^CAC^, Asp^GAC^	Probably yes
3	PF3D7_0202400	Gamete antigen 27/25, putative (translation enhancing factor)	0.55	1.17	−1.46	No	No	15	PF3D7_1310800	Uncharacterized protein	−0.51	−0.62	0.10	No	Yes
4	PF3D7_1368100	26S proteasome regulatory subunit RPN11, putative	0.55	1.71	−0.45	Asp^GAT^	Yes	16	PF3D7_1357000	Elongation factor 1-alpha	−0.52	−1.54	n.d.	His^CAC^, Asp^GAC^	Probably yes
5	PF3D7_1208600	Mitochondrial import inner membrane translocase subunit TIM10, putative	0.55	2.26	−0.92	His^CAT^	Probably yes	17	PF3D7_1453700	Cochaperone p23	−0.53	−1.77	2.09	His^CAC^	Unclear
6	PF3D7_1312500	Uncharacterized protein	0.54	2.26	n.d.	His^CAT^	No	18	PF3D7_1408600	40S ribosomal protein S8	−0.55	−1.54	2.77	His^CAC^	No
7	PF3D7_1140100	V-type proton ATPase subunit F	0.52	1.25	−1.22	No	Unknown	19	PF3D7_0415900	Ribosomal protein L15	−0.61	−0.76	0.42	His^CAC^	Yes
8	PF3D7_0917900	Heat shock protein 70 (BIP)	0.52	1.11	−1.24	His^CAT^	Probably yes	20	PF3D7_0802000	Glutamate dehydrogenase	−0.62	−0.60	0.10	No	Probably no
9	PF3D7_1343700	Kelch protein K13	0.51	1.28	−0.77	Asp^GAT^	Yes	21	PF3D7_1317800	40S ribosomal protein S19	−0.64	−1.77	0.33	No	Yes
10	PF3D7_0831700	Heat shock protein 70	0.51	2.07	−0.01	Asp^GAT^	No	22	PF3D7_1321200	Uncharacterized protein	−0.65	−0.76	1.95	No	Yes
11	PF3D7_1371900	Uncharacterized protein	0.50	0.99	−0.02	His^CAT^	Probably no								
12	PF3D7_0822100	Mediator of RNA polymerase II transcription subunit 7	0.50	1.87	−0.11	Asp^GAT^	Probably yes								

The numbers correspond to proteins in Fig. 3c. His and Asp codon biases are shown in Extended Data Fig. 3b,c and Supplementary Tables 5–8. Essentiality was determined by transposon mutagenesis^90^, as listed in PlasmoDB. n.d., not determined.

We analysed the GO slim and PlasmoDB databases for protein functionality and essentiality, respectively. For Lys^AAA^-enriched upregulated proteins, top functional terms included ‘unfolded protein response’ and ‘ATP dependent activity’. For the Lys^AAG^-enriched downregulated proteins, top GO slim terms included RNA binding and ribosome structural components (Fig. 3e, Extended Data Fig. 3d and Supplementary Tables 3 and 4). Three of the downregulated proteins displayed codon bias for Lys, His and Asp, suggesting that these proteins may have a regulatory role in the DHA-induced stress response. This included a 60S ribosomal protein (Pf3D7_1142500), an inner membrane complex subcompartment protein (Pf3D7_1460600) and the conserved translation factor eEF1-α^64^.

**Extended Data Fig. 4:**
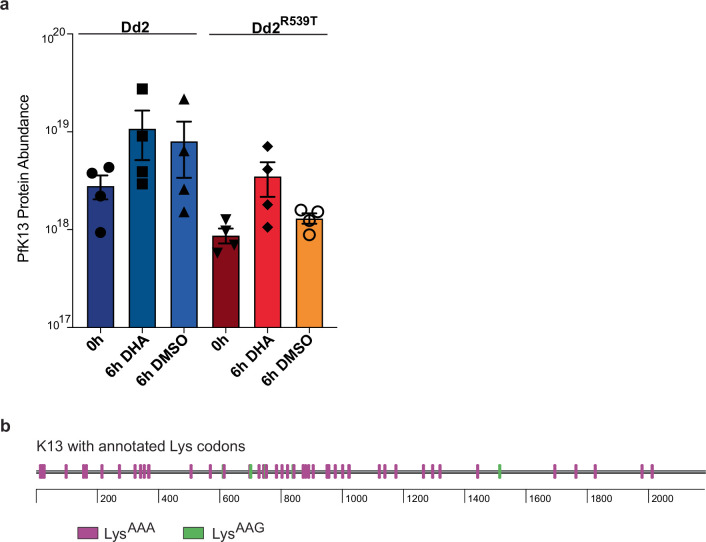
K13 is a highly Lysine AAA codon-biased protein whose levels increase after dihydroartemisinin treatment in Dd2^R539T^ parasites. (**a**) Analysis of TMT quantified protein levels from proteomics experiments (Fig. 2; Source Data Extended Data Fig. 4). Protein abundance is shown at 0 and 6 h post either DHA or DMSO treatment in Dd2 or Dd2^R539T^. Individual bioreplicates (*n* = 4) are shown, with error bars representing ± s.e.m. (**b**) Lysine codon usage in the K13 protein. Coding sequence was analyzed in Seqbuilder Pro. Every lysine codon in the protein was analyzed for the use of Lys^AAA^ or Lys^AAG^. Source data

Several upregulated proteins in our Dd2^R539T^ parasites demonstrated bias for at least two codons (Supplementary Table 9). Most striking was K13, which was upregulated translationally, downregulated transcriptionally and had a codon bias for both Lys and Asp. By quantifying protein levels (based on TMT proteomics), we observed decreased PfK13 levels in Dd2^R539T^ parasites compared with Dd2, as previously reported^15,65^. Interestingly, for Dd2^R539T^, K13 protein levels increased in DHA-treated parasites while remaining unchanged upon DMSO treatment (Extended Data Fig. 4a). For K13, 52 of 57 lysine codons were Lys^AAA^, which clustered mostly in the first half of the gene (Extended Data Fig. 4b). These data suggest that K13 levels may be modulated by this codon-biased translational mechanism, providing a means for a rapid increase as parasites prepare to exit DHA-induced quiescence.

### Pf3D7_1019800 (PfMnmA) is required for parasite development

tRNA s^2^U modifications are known to regulate protein levels of Lys codon-biased proteins in yeast^44^, creating a mechanistic correlation between our tRNA and proteomic observations of DHA-treated ART-R parasites. Further support for a role of this pathway in ART resistance came from (1) previous transcriptomic data, which demonstrated that three genes in the s^2^U biosynthesis pathway were significantly over-represented (*P* = 0.003) in DHA-treated Dd2^R539T^ parasites, namely a putative tRNA 2-thiouridylase (PF3D7_1019800, PfMnmA), an aminomethyltransferase (PF3D7_134000) and a GTPase (PF3D7_0817100)^23^; (2) U_34_ s^2^U modification changes that are linked to translational fidelity and amino acid homeostasis^49,66–68^ and (3) U_34_ s^2^U hypomodification that leads to translational stalling on Lys^AAA^ codons, which in yeast causes a substantial growth slowdown^44^.

**Fig. 4:**
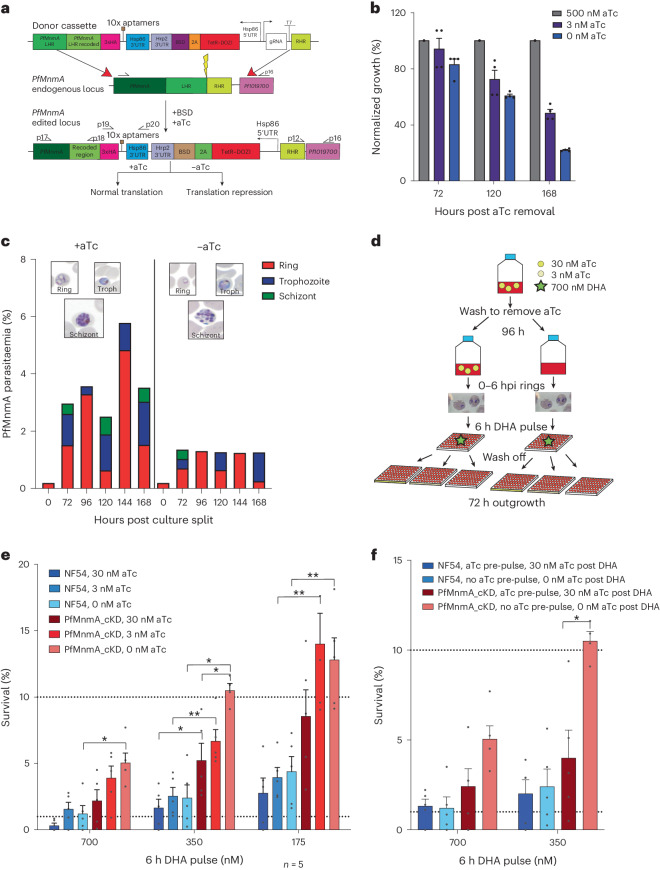
Knockdown of PfMnmA, the terminal thiouridylase in s^2^U biosynthesis, leads to increased ART survival. **a**, A schematic of donor plasmid PSN054, the endogenous *Pf3D7_1019800* (*PfmnmA*) locus and the recombinant locus of the edited cKD parasite. +aTc, normal translation and −aTc, protein knockdown. Edited parasites were confirmed via PCR and western blot analyses (Extended Data Fig. 5a,b). UTR, untranslated region; BSD, blasticidin S deaminase; LHR, left homology region. **b**,**c**, Synchronized, ring-stage PfMnmA_cKD parasites were washed to remove aTc and assayed in parallel with NF54 parasites. Washed parasites were inoculated in high (500 nM), low (3 nM) or no (0 nM) aTc and growth was followed by flow cytometry (**b**). Data were normalized to high aTc parasitaemias and represented as a percentage of growth. *n* = 5 independent biological replicates. The error bars represent ±s.e.m. Washed parasites were cultured ±aTc. Thin smears were Giemsa stained and 100 RBCs were counted (**c**). The *y* axis shows total parasitaemias (Extended Data Fig. 5d). **d**, A schematic of the modified RSA. Parasites were cultured with aTc, washed 3× and split into cultures ±aTc for 96 h. Synchronized, early ring-stage parasites (0–6 hpi) were exposed to a 6 h pulse of a range of DHA concentrations, the drug was washed off and then allowed to recover in 30 nM, 3 nM or 0 nM aTc for 72 h. **e**, RSA survival rates for NF54 and PfMnmA_cKD parasites cultured −aTc for 96 h before DHA exposure and allowed to recover on 30 nM, 3 nM or 0 nM aTc for 72 h. The results demonstrate the percentage of parasites that survived a range of DHA concentrations (≤700 nM aTc) relative to no-drug control parasites assayed in parallel. Percent survival values are shown as means ± s.e.m. **f**, RSA survival rates for parasites without MnmA knockdown (maintained with aTc) and with MnmA knockdown (maintained without aTc) exposed to 700 nM and 350 nM DHA for 6 h. *n* = 5 independent biological replicates. Statistical significance was determined via two-tailed Mann–Whitney *U*-tests as compared with the isogenic line or for the knockdown ±aTc. *P < 0.05 and **P < 0.01 (Source data and Extended Data Fig. 8). Source data

**Extended Data Fig. 5:**
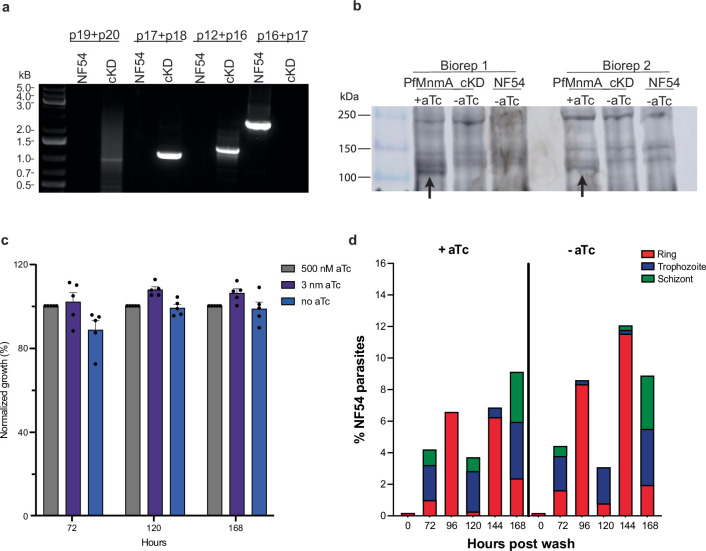
Validation of PfMnmA_cKD construct. CRISPR/Cas9 gene editing was used for creation of the PfMnmA_cKD line as shown in Fig. 4a. (**a**) Four sets of PCRs were used to confirm editing with primers listed in Supplementary Table 10 and shown in Fig. 4a. (**b**) Western blots probed with anti-HA antibody to confirm loss of MnmA protein expression. PfMnmA_cKD parasites were cultured ± aTc for 96 h prior to harvesting ABS parasites. Parental NF54 parasites were cultured without aTc and harvested in parallel. The arrow points to PfMnmA (130.5 kDa). Top bands represent internal loading controls resulting from HA-antibody cross-reacting proteins. Two independent biological replicates are shown. (**c**) aTc has no effect on NF54 parasite growth. Highly synchronized, ring-stage NF54^attB^ parasites were inoculated to the same parasitaemia in high (500 nM), low (3 nM) and no (0 nM) aTc. Growth was followed by flow cytometry (see Supplementary Methods) and experiments were run in parallel with PfMnmA_ckD parasites (Fig. 4b). Data were normalized to the parasitaemia at high aTc and represented as a percentage of growth at 3 nM aTc or 0 nM aTc. *n* = 5 independent biological replicates. Error bars represent SEM. (**d**) aTc has no effect on NF54 parasite progression through the asexual blood stage life cycle. NF54 was cultured ± aTc. At each of the indicated time points, thin smears were Giemsa stained and 100 RBC were counted (Source Data Fig. 4). Parasites were divided into ring, trophozoite and schizont stages with total parasitaemia shown on the y-axis. NF54 parasites were processed in parallel with PfMnmA_cKD (Fig. 4c). *n* = 1 biological replicate. Source data

To test the potential contribution of the U_34_ s^2^U modification, we generated a conditional knockdown (cKD) of PfMnmA (Pf3D7_1019800). This gene was selected as its product catalyses the terminal step in s^2^U biosynthesis in bacteria and eukaryotic mitochondria (in the eukaryotic cytosol the Ncs6–Urm1 pathway is used)^47^. In our dataset, PfMnmA was differentially regulated in Dd2^R539T^, but not Dd2 parasites, after DHA exposure^23^. To generate this cKD, we used the TetR–DOZI system that uses anhydrotetracycline (aTc) to regulate translation^69^ (Fig. 4a). Translation occurs in the presence of aTc, whereas removal leads to translation repression. cKD parasites were generated in an NF54 (ART-S) line that constitutively expresses the T7 polymerase and Cas9 (referred to as NF54 below)^69^. Successful creation of NF54_PfMnmA_cKD parasites (referred to below as PfMnmA_cKD) was confirmed using PCR, Sanger sequencing and western blot analysis (Extended Data Fig. 5a,b and Supplementary Results).

**Extended Data Fig. 6:**
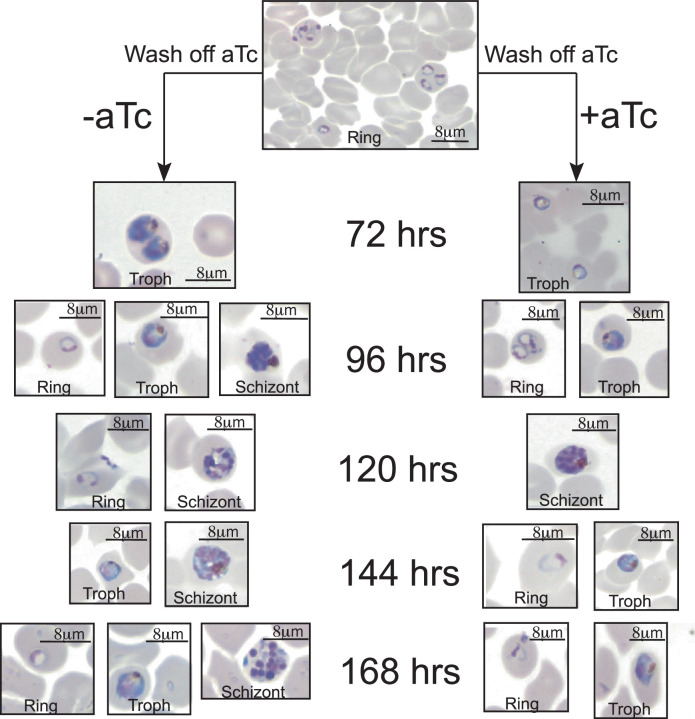
PfMnmA_cKD parasites cultured without aTc show defective schizonts. PfMnmA cultures were thoroughly washed and cultured ± aTc (Fig. 4c). At each of the indicated time points, thin smears were Giemsa stained. Images are representative of ring, trophozoite and schizont morphology noted at these times. Defects in schizont development appeared as early as 120 h. *n* = 1 biological replicate.

**Extended Data Fig. 7:**
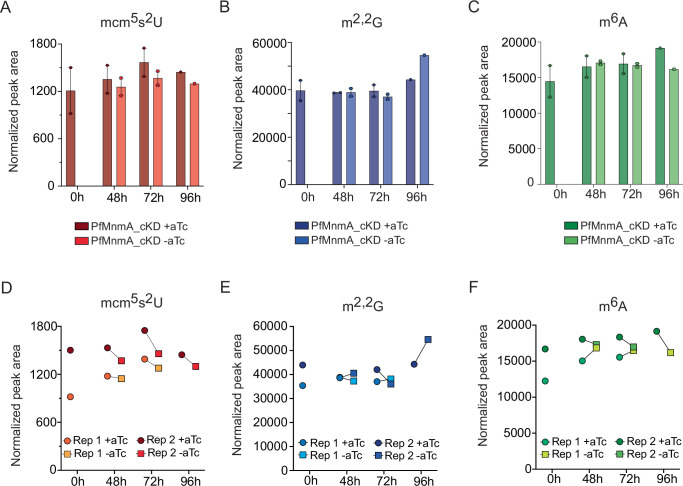
Knockdown of PfMnmA leads to decrease in mcm^5^s^2^U modification levels but not in levels of m^6^A or m^22^G modifications. Highly synchronized PfMnmA_cKD parasites were initially cultured with 500 nM aTc, thoroughly washed, and split into cultures with 500 nM (translation on) or 0 nM (translation off) aTc. The experiment was started with trophozoites. Parasites were harvested at 0, 48 and 96 h after aTc removal. Cultures were also begun with synchronized rings and harvested these at 72 h after aTc removal to recover an additional trophozoite sample. Control cultures propagated with aTc were harvested in parallel. Changes in the relative quantities of **(a)** mcm^5^s^2^U, **(b)** m^22^G, or **(c)** m^6^A modifications were quantified by LC-MS/MS in total tRNA extracted from parasites at the indicated time points, for +aTC or -aTc. Values are shown as means ± s.e.m. Data for each individual replicate and parasite pair are shown for **(d)** mcm^5^s^2^U, **(e)** m^2,2^G, or **(f)** m^6^A to demonstrate trends between replicates. *n* = 2. For the 96 h time point we have a single replicate (with technical duplicates), as the RNA was degraded during the extraction process in replicate 1. Statistical significance was determined via two-tailed paired *t*-tests. p<0.05 for mcm^5^s^2^U modification changes ± aTc and p>0.1 for m^22^G and m^6^A modifications changes ± aTc (Source Data Extended Data Fig. 7). Source data

Growth studies showed that PfMnmA_cKD parasites cultured in low (3 nM) or no aTc displayed a slow onset of death as compared with parasites cultured with high aTc (500 nM). NF54 parasites had no change in growth (Fig. 4b and Extended Data Fig. 5c). cKD parasites grown without aTc had evident defects in schizont morphology (Fig. 4c, Extended Data Figs. 5d and 6, Supplementary Fig. 1 and Supplementary Results). LC–MS/MS evaluation of global mcm^5^s^2^U modifications in PfMnmA_cKD parasites ±aTc revealed specific decreases in total levels of mcm^5^s^2^U in parasites grown without aTc, as compared with those grown with aTc. There were no changes, however, in m^2,2^G or m^6^A (Extended Data Fig. 7 and Supplementary Results). These findings suggest that the PfMnmA knockdown leads to specific decreases in global mcm^5^s^2^U modification levels, although this does not abolish the modification fully, probably because of the cytosolic s^2^U biosynthetic pathway.

### Knockdown of MnmA results in increased resistance to ART

We predicted that if s^2^U hypomodification and its downstream consequences contribute to ART resistance, then a PfMnmA knockdown should have decreased ART sensitivity. To test this, we modified the RSA to incorporate the growth kinetics of our cKD line (Fig. 4d and Methods). At 96 h before drug exposure, parasites were washed and split into media ±aTc. On the day of the assay, highly synchronized early rings (0–6 hpi) were pulsed for 6 h with DHA (concentration range: 700 nM to 1.4 nM), washed and allowed to recover for 72 h in the presence of 30 nM, 3 nM or 0 nM aTc. Parasites maintained on 30 nM aTc before and after DHA comprised the ‘translation on’ control. Parasites cultured without aTc before and after DHA constituted the ‘translation off’ control. Chloroquine (CQ) was used as an unrelated control.

**Extended Data Fig. 8:**
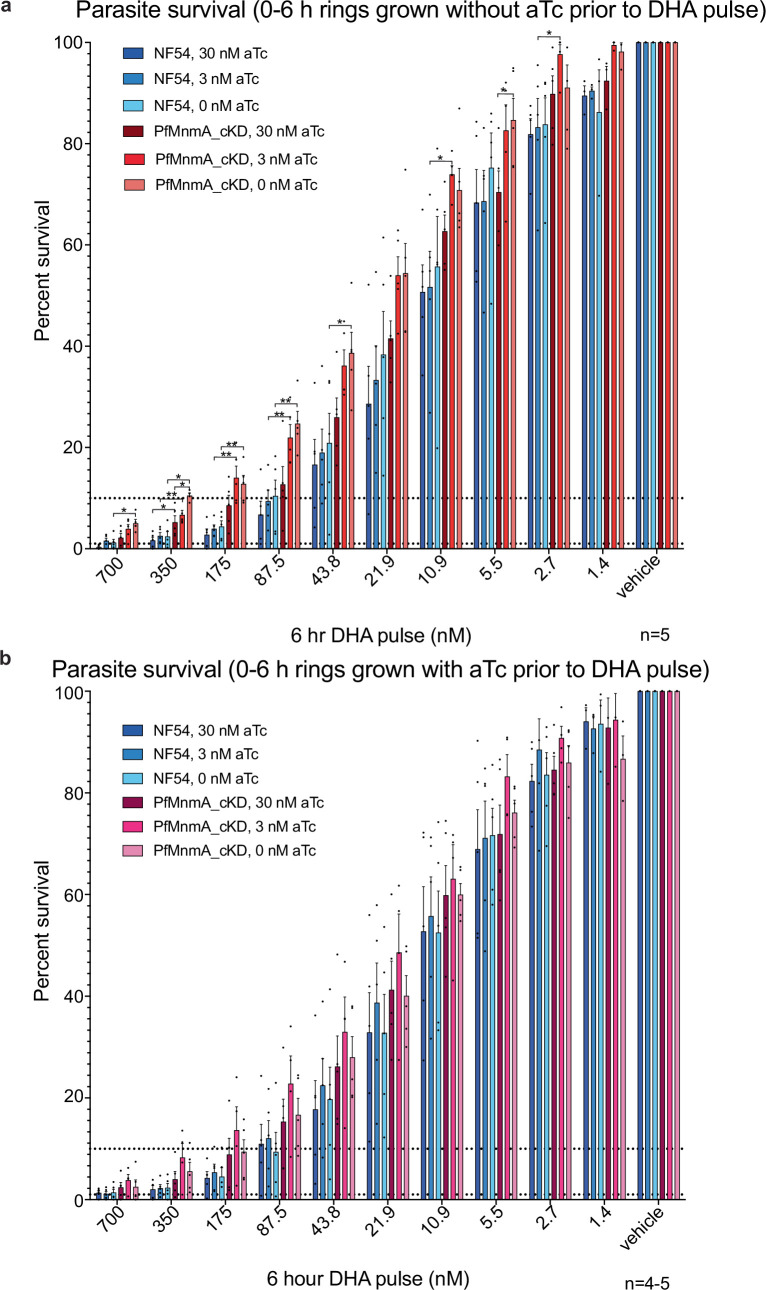
Ring-stage survival assays demonstrating that knockdown of PfMnmA for 96 hr prior to dihydroartemisinin (DHA) pulse leads to increased resistance, while knockdown after DHA pulse has no effect. RSAs were performed as outlined in Fig. 4d. Highly synchronized 0-6 h ring stage parasites cultured for 96 h without aTc (**a**) or with aTc (**b**) prior to drug pulse were exposed to a 6 h pulse of a range of DHA concentrations, drug was washed off 3× and parasites were allowed to recover on plates with 30 nM aTc, 3 nM aTc or 0 nM aTc for 72 h. Results demonstrate the percentage of early ring-stage parasites (0-6 hpi) that survived a range of DHA concentrations beginning at 700 nM aTc relative to no-drug control parasites assayed in parallel. Percent survival values are shown as means ± s.e.m. *n* = 5 independent biological replicates. Statistical significance was determined via two tailed Mann Whitney *U* tests as compared to the isogenic line or for the knockdown ± aTc. *p< 0.05 and **p< 0.01 (Source Data Extended Data Fig. 8 and Supplementary Fig. 2). Source data

PfMnmA_cKD parasites that underwent protein knockdown (no aTc for 96 h) before DHA exposure demonstrated an aTc concentration-dependent increase in ART survival, as compared with NF54 (Fig. 4e). At both 700 and 350 nM DHA, NF54 parasites did not survive. However, PfMnmA_cKD parasites cultured on 30, 3 or 0 nM aTc post-DHA pulse survived significantly more than NF54 controls (*P* < 0.05%). At 350 nM DHA, survival differences were more pronounced with an aTc concentration-dependent increase in survival, evident at DHA concentrations as low as 2.7 nM in PfMnmA_cKD parasites (Extended Data Fig. 8a and Supplementary Results).

PfMnmA parasites cultured on 30 nM aTc before the DHA pulse (that is, with translation on) demonstrated no significant differences in survival as compared with DMSO controls (Extended Data Fig. 8b). These data suggest that protein knockdown is essential before DHA exposure to prepare the parasites for this response. There were no differences in survival after CQ exposure for any condition (Supplementary Fig. 2a,b).

To confirm that our phenotype was secondary to PfMnmA knockdown, we compared our ‘translation off’ and ‘translation on’ parasites. At 350 nM ART, the former had significantly more survival than the latter (11% versus 4% survival, *P* < 0.05), further suggesting that decreased levels of MnmA led to increased ART survival (Fig. 4f). These experiments supported the importance of s^2^U tRNA hypomodification in the ART-induced stress response.

### MnmA knockdown parasites show altered anti-malarial susceptibility

**Fig. 5:**
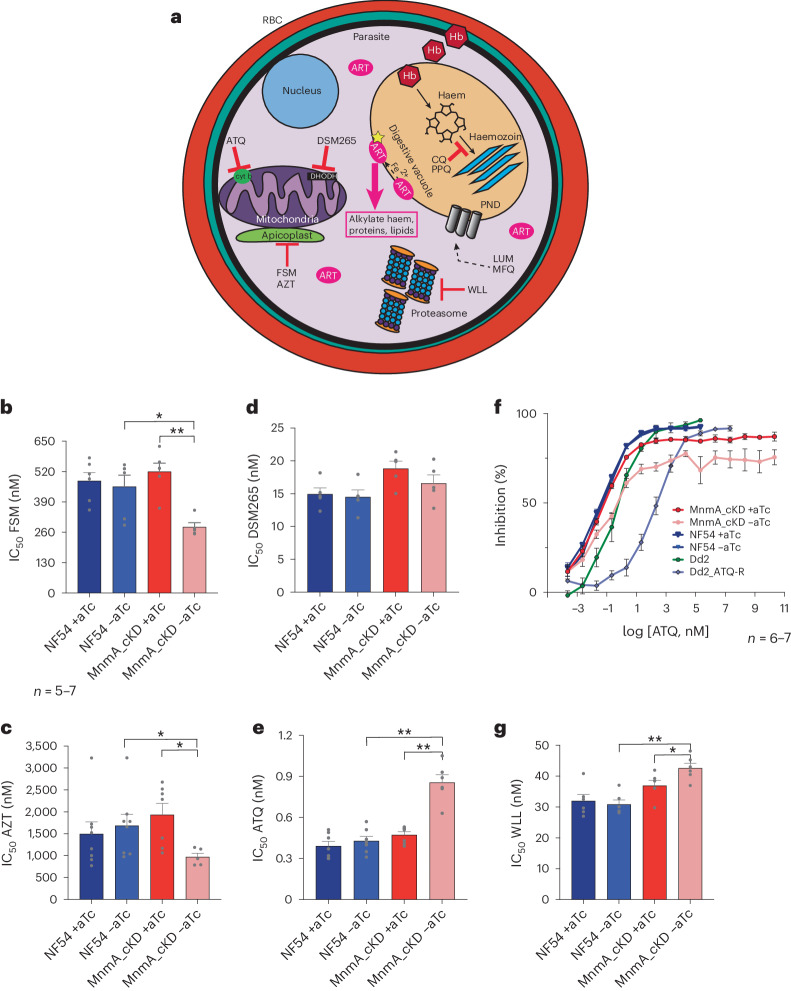
PfMnmA contributes to parasite responses to multiple stressors. **a**, A schematic of molecular sites of action for anti-malarials used in this study. Hb, haemoglobin; LUM, lumefantrine; MFQ, mefloquine; PPQ, piperaquine. **b**–**e**,**g**, IC_50_ data shown as means ± s.e.m. from 72 h dose–response assays of asynchronous NF54 parasites ±aTc, PfMnmA parasites cultured with aTc and PfMnmA parasites cultured without aTc for 96 h before drug pulse (Extended Data Fig. 9a) for FSM (**b**), AZT (**c**), DSM265 (**d**), ATQ (**e**) and WLL (**g**). *n* = 5–7. Statistical significance was determined via two-tailed Mann–Whitney *U*-tests. *P < 0.05 and **P < 0.01. **f**, Dose–response curves for ATQ for NF54 parental line with and without aTc, PfMnmA parasites cultured with aTc and PfMnmA parasites cultured without aTc for 96 h before drug pulse, and Dd2 and Dd2_ATQ-R (ATQ resistant, Dd2-CYT1-V259L). The error bars represent s.e.m. *n* = 6–7 independent biological replicates per parasite line. Source data

**Extended Data Fig. 9:**
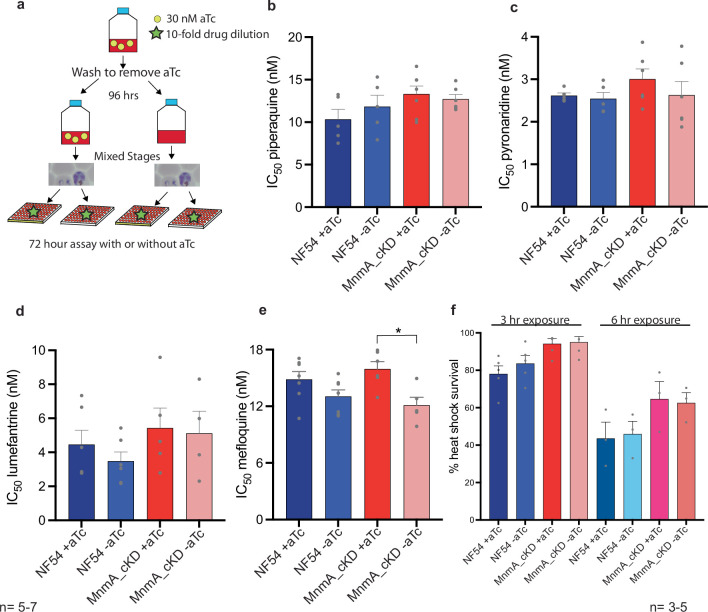
IC_50_ data shown as mean ± SEM from 72 h dose–response assays of asynchronous NF54 and PfMnmA knockdown parasites. (**a**) Schematic of 72 h growth assay set up. NF54 parasites and PfMnmA parasites were washed 96 h prior to experiment and split into 30 nM or 0 nM aTc cultures. For experiment, parasites were washed and cultured with (30 nM) or without (0 nM) aTc and (**b**) piperaquine, (**c**) pyronaridine, (**d**) lumefantrine, and (**e**) mefloquine. *n* = 5 to 7 independent biological replicates per parasite line. Statistical significance was determined via two-tailed Mann Whitney *U* tests. *p<0.05, **p<0.01. (**f**) Heat shock survival for highly synchronized trophozoite (26-34 hpi) NF54 parental parasites ± aTc, PfMnmA_cKD parasites cultured with aTc, and PfMnmA_cKD parasites cultured without aTc for 96 h prior to exposure to either 37 °C or 42 °C for 3 or 6 h. Percent survival is compared to the control parasite sample that did not undergo heat shock (Source Data Extended Data Fig. 9). Error bars are s.e.m. *n* = 3 to 5 independent biological replicates per parasite line. Source data

We next addressed whether PfMnmA knockdown would affect parasite susceptibility to other anti-malarials. Parasites were cultured for 96 h (±aTc) and then exposed to twofold serial dilutions of drug for 72 h ±aTc (Extended Data Fig. 9a). We tested three groups of compounds. The first group contained the apicoplast-targeting compounds azithromycin (AZT) and fosmidomycin (FSM), which were selected based on recent data showing that PfMnmA is necessary for apicoplast maintenance^70^. Knockdown of MnmA led to low-level but significant twofold sensitization to both compounds, as compared with non-knockdown conditions (Fig. 5b,c and Supplementary Results).

The second group contained the mitochondrial inhibitors DSM265 and atovaquone (ATQ), which were selected because eukaryotic homologues of PfMnmA have been localized to the mitochondria^47^. While no change was observed for the DHODH inhibitor DSM265 (Fig. 5d), PfMnmA knockdown led to a 1.8-fold increase in the half-maximum inhibitory concentration (IC_50_) of the cytochrome bc1 inhibitor ATQ (Fig. 5e). Knockdown parasites never showed >90% killing, suggesting a small population of ATQ-tolerant parasites (Fig. 5e,f). This profile differed from ATQ-resistant control CYTb V259L parasites that showed >100-fold increases in IC_50_ and 90% of the maximum inhibitory concentration values relative to the Dd2 parent (Fig. 5f and Supplementary Results).

The third group contained compounds that are known (lumefantrine (LMF), piperaquine, mefloquine (MFQ) and pyronaridine (PND)) or potential (WLL) ART-based combination therapy partner drugs with differing mechanisms of action^71–74^(Fig. 5a). The *P.* *falciparum* response to ART derivatives has been linked to the ubiquitin–proteasome system, the unfolded protein response and heat shock, and is related to ART-induced proteotoxic stress^19,31,75–77^. Knockdown parasites in 0 nM aTc showed a small but significant increase in their mean IC_50_ value with the proteasome inhibitor WLL, as compared with non-knockdown conditions (Fig. 5g). In contrast, no changes in survival were seen when parasites were exposed to 42 °C for 3 or 6 h (ref. ^76^) (Extended Data Fig. 9f and Supplementary Results). Our data indicate that despite overlaps among ART resistance, proteotoxic stress and the heat shock response, the underlying mechanisms are more nuanced. No significant differences were noted in PfMnmA_cKD parasites after aTc removal in response to piperaquine, PND, MFQ or LMF (Extended Data Fig. 9b–e, Supplementary Fig. 3a–h and Supplementary Results).

## Discussion

We describe an epitranscriptomic stress-response mechanism in ART-R *P.* *falciparum* parasites, whereby reprogramming of thiouridine tRNA modifications can modulate the response to ART exposure. Combining mass spectrometry, proteomics and genetic knockdowns, we identify a role for thiouridine tRNA modifications in resistance to ART and other drug stressors. We provide evidence that K13 can be regulated by codon bias translation upon removal of ART pressure. These findings implicate a previously unrecognized role for epitranscriptomic mechanisms in modulating *P.* *falciparum* susceptibility to drug-induced stress.

tRNA modification reprogramming dynamically regulates cellular adaptation to environmental perturbations, including nutrient availability and exogenous stressors^41,49,50,60,78^. Recent work has identified a role for tRNA modification reprogramming in ABS parasite development^62^. Here, we provide compelling evidence that dynamic reprogramming of tRNA modifications, specifically mcm^5^s^2^U, exists in ART-R parasites in response to drug-induced stress (Supplementary Discussion). An analogous situation exists in melanoma cells, whereby alterations in tRNA modifications can contribute to chemotherapy resistance^79^.

**Extended Data Fig. 10:**
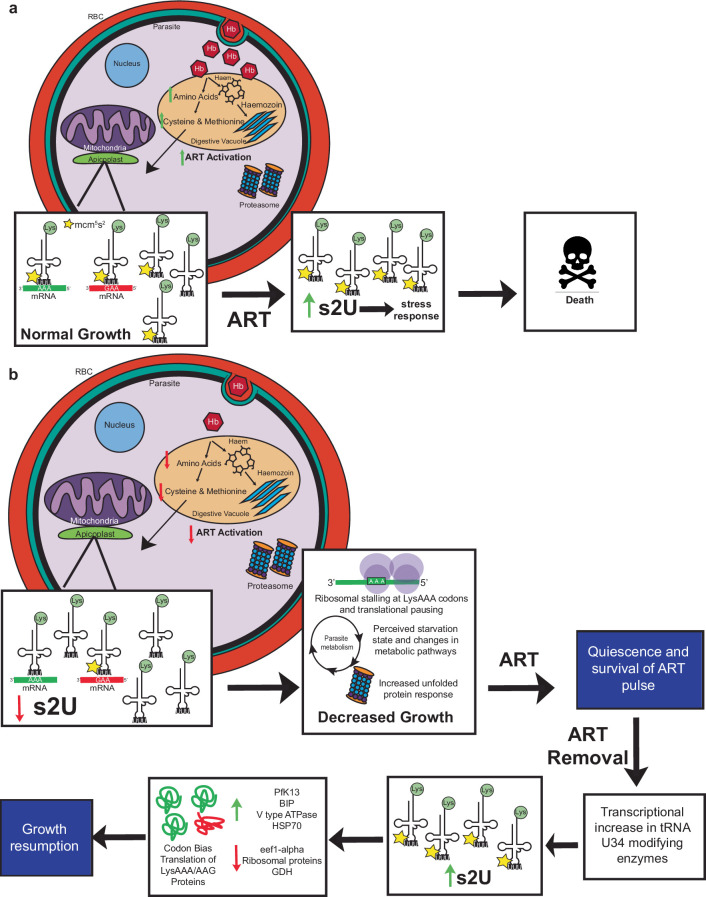
Working model for the role of s^2^U tRNA modification reprogramming in resistance to artemisinin in both ART-sensitive and -resistant parasites. (**a**) Sensitive, K13-wild-type parasites have normal levels of hemoglobin endocytosis leading to normal levels of hemoglobin-derived peptides and amino acids^14,15^, presumably including cysteine and methionine. This enables normal thiouridine modifications and normal parasite growth. In response to artemisinin pressure, s^2^U modifications increase (Fig. 1b, c); however, the overwhelming cellular stress leads to all parasites dying. (**b**) Artemisinin-resistant, K13 R539T parasites have reduced hemoglobin endocytosis and hemoglobin-derived peptides^14,15^, likely leading to reduced availability of sulfur-containing amino acids cysteine and methionine. Parasites sense decreased levels leading to s^2^U hypomodification (Fig. 1b, c)^64–66^. This results in decreased parasite growth, possibly via ribosomal stalling on -A ending Glu, Gln and Lys codons, reprogramming of parasite metabolism, or both. s^2^U hypomodification also leads to increased levels of proteotoxic stress and increases in the unfolded protein response (Fig. 2c). This enables a subpopulation of parasites to survive ART pulse. At 6 h post drug pulse, enzymes in the U_34_ thiol modification pathway, including PfMnmA are transcriptionally upregulated^22^. This likely increases s^2^U modifications, which results in Lys codon-biased translation in the post-ART parasite proteome (Fig. 3). Subsequent upregulation of Lys^AAA^ enriched proteins, including K13 and its interacting partner BIP, may enable rapid increases in K13 protein levels required for parasites to resume growth (Extended Data Fig. 4). Lys^AAA^ enriched proteins also include members of the unfolded protein response. Downregulated Lys^AAG^-enriched proteins include ribosomal proteins and the eukaryotic elongation factor eef1-α. Together these alterations may prime the subpopulation of surviving parasites for growth after ART removal.

Decreased haemoglobin endocytosis is central to mutant K13-mediated ART resistance^15,16,21^. This leads to decreased ART activation due to less available Fe^2+^-haem and to decreased amino acid availability secondary to reduced availability of haemoglobin-derived peptides^23^. In yeast, mcm^5^s^2^U is one of the few tRNA modifications that links translation with nutritional status^66–68^. By sensing availability of the sulfur-containing amino acids cysteine and methionine, mcm^5^s^2^U reprogramming leads to decreased carbohydrate metabolism, translation and growth. These features are also noted in quiescent, ART-R *P.* *falciparum* parasites^20,25,30,32,33^. It is tempting to hypothesize that decreased levels of available methionine in K13-mutant parasites post-DHA exposure^80^ can lead to s^2^U hypomodification (Extended Data Fig. 10b). A second but not mutually exclusive hypothesis is that s^2^U hypomodification can enhance the unfolded protein response, a core feature of ART resistance (Supplementary Discussion)^19,23,75^.

In ART-R parasites, we identified proteins regulated by codon bias translation, including 12 upregulated and 10 downregulated proteins for Lys. K13 had significant Lys^AAA^ codon bias, as did its interactor BIP (Pf3D7_0917900) (Supplementary Discussion). K13 also displayed codon bias for Asp^GAT^. Our proteomics data confirmed reduced K13 levels in Dd2^R539T^ parasites^15,65^. Unexpectedly, K13 mutant parasites increased their K13 levels after DHA, but not DMSO, treatment. In contrast, ART-S parasites increased K13 levels equally after both. *k13* harbours a high concentration of clustered Lys^AAA^ codons compared with cognate Lys^AAG^ ones. Our data suggest that K13 levels, modulated by codon bias translation, rise as ART-R parasites exit quiescence and allow for increased haemoglobin endocytosis and growth resumption (Extended Data Fig. 10b). Codon bias in *k13* might reflect its central role in regulating haemoglobin endocytosis and intracellular redox states^15,16,23,81,82^.

To test the role of s^2^U hypomodification in ART resistance, we created a cKD of the terminal enzyme (MnmA) in s^2^U biosynthesis. This pathway is highly conserved across prokaryotes and eukaryotic mitochondria^83^. A second pathway for s^2^U modification exists in the cytosol of eukaryotic organisms, with Ncs6 serving as the terminal thiouridine synthetase^48^. Despite the PfMnmA organellar localization, we observed a specific decrease in the global levels of the mcm^5^s^2^U modification in PfMnmA_cKD parasites. In yeast, mitochondrially produced sulfur species are exported into the cytosol and required for cytosolic tRNA thiolation^84,85^. Disruption of the thiolation pathway in the apicoplast may similarly alter cytosolic tRNA thiolation. Given that mcm^5^s^2^U modifications were not fully ablated in PfMnmA_cKD parasites, our data support the existence of a cytosolic thiouridine synthesis pathway in *P.* *falciparum*^86^.

In other organisms, mcm^5^s^2^U modifications have been implicated as modulators for a multitude of perturbations, including heat, oxidative and endoplasmic reticulum stresses^40,47,78,87^. In *P.* *falciparum*, studies have suggested overlaps between parasite responses to ART, fever and oxidative and proteotoxic stresses^23,75,77^. Apicoplast pathways were implicated in both the ART-R response to DHA^23^ and the ART-S response to heat shock^77^. Our PfMnmA knockdown displayed increased ART survival, confirming our hypothesis that s^2^U hypomodification plays a role as an epitranscriptomic modifier of parasite survival to ART. We also found a small, but statistically significant, decrease in susceptibility to the proteasome inhibitor WLL in our translationally repressed PfMnmA_cKD parasites, but no difference in heat shock survival. We also unexpectedly uncovered an ATQ tolerance phenotype (Supplementary Discussion). Our study highlights the possibility that regulatory pathways in the apicoplast may affect translation of cytosolic proteins in response to stress by altering the synthesis of tRNA modifications involving a thiouridine such as s^2^U.

In conclusion, we have identified a role for tRNA thiouridine modification reprogramming in ART resistance and stress responses in *P.* *falciparum*. We propose the following working model (Extended Data Fig. 10): in ART-S parasites, haemoglobin endocytosis leads to an abundance of amino acids and haem and normal mcm^5^s^2^U modifications of Lys tRNAs. Parasites can increase mcm^5^s^2^U modifications in response to ART stress, but this cannot overcome drug-induced cellular damage (Extended Data Fig. 10a). In contrast, decreased levels of haemoglobin endocytosis in ART-R parasites lead to decreased amino acid levels and hypomodification of tRNAs, including mcm^5^s^2^U. In turn, these parasites decelerate translation, alter metabolism and have a chronic level of proteotoxic stress. This is highly adaptive to short-acting ART-induced stress, despite being maladaptive in other settings. After drug exposure, upregulation of the s^2^U modification biosynthesis pathway leads to increased levels of s^2^U modifications, which produce the Lys codon bias that we observed in the post-DHA proteome. Codon bias-regulated proteins, including K13 that shows increased levels, respond to the changing cellular conditions, and can lead to growth resumption (Extended Data Fig. 10b). Our findings open an unexplored area of research by identifying how drug-resistant parasites employ differential epitranscriptomic stress response mechanisms as a means of survival.

## Methods

### Synchronization and sampling of parasite culture

ART-S Dd2 parasites (expressing the WT *k13* allele with silent binding-site mutations) and its isogenic, ART-R Dd2^R539T^ derivative parasite line, which had been gene edited to express the K13 R539T mutation^7,12,23^, were cultured as previously described^88^. *P.* *falciparum* ABS parasites were cultured in human erythrocytes (Interstate Blood Bank) at 3% haematocrit in RPMI-1640 medium supplemented with 2 mM l-glutamine, 50 mg l^−1^ hypoxanthine, 25 mM HEPES, 0.21% NaHCO3, 10 mg l^−1^ gentamycin and 0.5% wt/vol Albumax II (Invitrogen). Parasites were maintained at 37 °C in 5% O_2_, 5% CO_2_ and 90% N_2_. Before the experiment start, lines were confirmed using Sanger sequencing of the *k13* locus. Lines were also checked for mycoplasma contamination using the e-Myco PLUS Mycoplasma PCR Detection Kit, as per the protocol. Parasites were synchronized to 0–6 hpi using both magnetic column purification (MACS Miltenyi Biotec) and 5% sorbitol. Briefly, trophozoites and schizonts were passed through the LD column (Miltenyi) and eluted by removal of the column from the magnet. The parasites were suspended into 3% haematocrit and allowed to reinvade for 14–16 h. Blood was pelleted and cultures were resuspended in 20 ml 5% sorbitol (Sigma-Aldrich) and incubated at 37 °C for 15 min to ensure only ring stages remained. At each culture expansion step, parasites underwent sorbitol treatment before expansion to maintain a high degree of synchronization. For each line, parasites were grown to at least 6% parasitaemia in 200 ml RPMI media with 3 ml packed RBC in T225 (Corning) flasks at 5% O_2_, 5% CO_2_ and 90% N_2_ gas. Dd2 and Dd2^R539T^ parasites were always assayed simultaneously to control for external conditions that may affect tRNA modifications. Parasites were evaluated before the experiment start to ensure that >85% of the culture was 0–6 hpi ring-stage parasites. If between 80% and 85% of the culture was the correct stage, then cultures underwent sorbitol synchronization to eliminate trophozoite and schizont stages as above. If cultures were less than 80% synchronized, the experiment was not performed. The concentration and duration of dihydroartemisinin pulse were chosen based on RSAs, as previously described^7^. At *t* = 0, parasites were collected for tRNA or proteomic analysis using saponin lysis. Briefly, infected RBCs were pelleted by centrifugation, washed with phosphate-buffered saline (PBS), incubated with 0.1% saponin for 10–15 min at 37 °C, pelleted by centrifugation, washed twice with PBS and parasite pellets were frozen at −80 °C for downstream analysis. For treated samples, the remaining culture was split and incubated with 700 nM DHA or DMSO vehicle. For tRNA modification analysis, samples were incubated for 6 h at 37 °C then collected by saponin lysis and frozen as described above. Smears of all flasks were made to assess per cent survival via staining with Giemsa staining and counting infected RBCs (Extended Data Fig. 1). For proteomic analysis, samples were pulsed with DHA or DMSO for 6 h, then washed three times with warm RPMI media and allowed to recover in T225 flasks at 37 °C until 12 h post initial DHA exposure. Parasites were then collected by saponin lysis and pellets frozen, as above.

A research protocol (IRB-AAAC4249) was submitted to the institutional review board at the Columbia University Irving Medical Center and was approved on 22 September 2022 by the institutional review board as ‘not human subjects research in accordance with the Code of Federal Regulations Title 45—Public Welfare Department of Health and Human Services, Part 46—Protection of Human Subjects’.

### LC–MS/MS identification of modified ribonucleosides in tRNA

Purified *P.* *falciparum* tRNA from seven biological replicates of selected timepoints were hydrolysed enzymatically as described previously^62^. A Hypersil GOLD a Q column (100 × 2.1 mm, 1.9 μm, Thermo Scientific) was used to resolve the digested ribonucleosides in a two-buffer eluent system, with buffer A consisting of water with 0.1% (vol/vol) formic acid and buffer B consisting of acetonitrile with 0.1% (vol/vol) formic acid. All solvents used were LC–MS grade. High-performance liquid chromatography (HPLC) was performed at a flow rate of 300 μl min^−1^. The gradient of acetonitrile with 0.1% (vol/vol) formic acid was as follows: 0–12 min, held at 0%; 12–15.3 min, 0–1%; 15.3–18.7 min, 1–6%; 18.7–20 min, held at 6%; 20–24 min, 6–100%; 24–27.3 min, held at 100%; 27.3–28 min, 100–0%; and 28–41 min, 0%. The HPLC column was directly connected to an Agilent 6490 triple quadrupole mass spectrometer with electrospray ionization Jetstream operated in positive ion mode. The voltages and source gas parameters were as follows: gas temperature 50 °C, gas flow 11 l min^−1^, nebulizer 20 psi, sheath gas temperature 300 °C, sheath gas flow 12 l min^−1^, capillary voltage 1,800 V and nozzle voltage 2,000 V.

### Protein extraction

The parasite pellet from three independent biological replicates was resuspended in 6× volume of 8 M urea containing 1 mM sodium orthovanadate and homogenized using a sonicator pulse for 3 min at 25% amplitude and 2 s on, 3 s off pulse time. The lysate was spun at 16,000*g* at 4 °C for 30 min to pellet the insoluble fraction and the lysate was transferred into a new tube. Protein (100 μg) was reduced with 10 mM dithiothreitol at 56 °C for 1 h and followed by reduction using 100 mM iodoacetamide for 1 h in the dark. This solution was diluted to 1 M urea and digested with 2 μg trypsin (Thermo Scientific) overnight at ambient temperature. The resulting peptides were desalted using Pierce desalting columns as per the manufacturer’s instructions. These peptides were reconstituted in triethylammonium bicarbonate and labelled using TMT labels (Thermo Scientific) as per the manufacturer’s instructions. The labelled peptides were combined, dried and reconstituted in 0.1% formic acid. After checking for labelling efficiency, these peptides were then separated into eight fractions using high-pH fractionation columns (Thermo Scientific). The labelling scheme is provided in Source data.

### LC–MS/MS analysis of the parasite proteome

Peptides were separated by reverse-phase HPLC (Thermo Scientific Easy nLC1000) using a pre-column (Thermo Scientific) and a self-pack 5 μm tip analytical column (15 cm of 5 μm C18, New Objective) over a 140 min gradient before nanoelectrospray using a QExactive HF-X mass spectrometer (Thermo Scientific). The mass spectrometer was operated in a data-dependent mode. The parameters for the full-scan MS were resolution of 70,000 across 350–2,000 *m****/****z*, AGC 3 × 10^6^ and maximum IT 300 ms. The full MS scan was followed by MS/MS for the top ten precursor ions in each cycle with a normalized collision energy of 28 (34 for TMT samples) and dynamic exclusion of 30 s. Raw mass spectral data files (.raw) were searched using Proteome Discoverer (Thermo Scientific) and Mascot version 2.4.1 (Matrix Science). Mascot search parameters were 10 ppm mass tolerance for precursor ions, 15 mmu for fragment ion mass tolerance, 2 missed cleavages of trypsin, fixed modification was carbamidomethylation of cysteine and variable modifications were lysine labelled TMT residues, peptide N-terminal TMT labels, methionine oxidation and serine, threonine and tyrosine phosphorylation. Only peptides with a Mascot score greater than or equal to 25 and an isolation interference less than or equal to 30 were included in the data analysis. For TMT samples, a minimum abundance of 500 ion counts was used as a threshold to ensure the robustness of data. Quantification and statistical testing of TMT proteomics data was performed using MSstats^89^.

### Data processing

Abundances of RNA modifications were normalized to canonicals rA, rU, rG and rC to account for total RNA amount injected. These were then transformed to log_2_ ratios of modification levels in each timepoint or dosage relative to either an arbitrary average or untreated control across all samples, respectively. Data analysis was performed in Excel (Microsoft). For interpretations of the relationships between codon usage (codon frequency) and upregulated and downregulated proteins at different timepoints, principal component regression was performed using Graphpad Prism. The values of codon usage in synonymous codon choices of those proteins were retrieved from the pre-calculated genome-wide codon usage as provided before^62^. The fold-change values from the proteomics data were used as input for the response variable in the principal component regression analysis. The codon usage was charted out as the predictors.

### Determination of proteins regulated by codon bias translation and their characteristics

To determine proteins regulated by codon bias translation, the top increased and decreased proteins in the Dd2^R539T^ parasite post-ART exposure were individually assessed for Lys, Asp and His codon usage. Calculated *z*-scores greater than 0.5 or less than −0.5 were used to delineate a bias in codons with enrichment of one codon seen in increased proteins and enrichment of the cognate codon noted in the decreased proteins (Supplementary Table 1 and Source data). To determine whether these proteins were post-transcriptionally regulated, we assessed the ratio of the fold‐change value for a protein to the fold‐change value for the corresponding mRNA at a specific timepoint from a previously published RNA sequencing Dd2^R539T^ dataset^23^. This represents an estimate of translational output per mRNA copy. Proteins were considered regulated by codon bias translation if they had a codon bias for Lys, His or Asp and the protein abundance (increased versus decreased) was opposite the transcriptional direction of the gene. Characteristics of codon bias proteins were determined by database search from PlasmoDB^90^. For functional characterization and putative localization, the top Malaria Parasite Metabolic Pathways and GO terms were selected for each protein. Predicted essentiality was determined from transposon mutagenesis and accessed via PlasmoDB^91^. The results were plotted in GraphPad Prism.

### cKD plasmid construction

A cKD regulated by aTc was created using clustered regularly interspaced short palindromic repeats (CRISPR)–Cas9 and the previously described PSN054 linear plasmid^69^. This plasmid contains the Tet repressor–DOZI helicase fusion regulatory component, 10x array 3′ RNA aptamers, blasticidin selection cassette and 3xHA tag. The plasmid also contains a guide RNA driven by the T7 promotor. The right homology region was amplified from Dd2 parasites with primers p12 and p16 (Supplementary Table 10). The left homology region was divided into two parts, with one part amplified from Dd2 parasites using primers p17 and p18 and the second part recodonized to *Saccharomyces cerevisiae* using a codon juggling algorithm^92^ and ordered from Integrated DNA Technologies. The right homology region was cloned into the IsCEI site via Gibson cloning (NEB). To maintain aptamers throughout, plasmids were transfected into Big TSAeasy cells (Lucigen) and grown on chloramphenicol Luria–Bertani agar plates at 30 °C. The left homology region (both native and recodonized) were then cloned in at the FseI and AlsI sites using Gibson cloning. Finally, two different guides were cloned into the AflII site via Gibson cloning. The plasmid underwent Sanger sequencing at each step to confirm insert insertion. The final plasmid was sequenced by Sanger sequencing and digested with XmaI to ensure aptamers remained intact. Plasmids were then grown in large volume cultures with chloramphenicol and arabinose at 30 °C and midi prepped before transfection.

### Creation of the Pf3D7_1019800_cKD line

NF54attB parasites that constitutively express Cas9 and the T7 RNA polymerase (referred to as NF54 from here on) were transfected as described^69^. The donor plasmid created above harbouring blasticidin *S*-deaminase was selected using 2 µg ml^−1^ blasticidin hydrochloride pressure until parasite recrudescence (Thermo Fisher). Cultures were maintained on 500 nM aTc at all times to ensure protein expression. Editing was confirmed using PCR primers p12, p16, p17, p18, p19 and p20 (Fig. 4a, Extended Data Fig. 5a and Supplementary Table 10) and Sanger sequencing, and clones were obtained by limiting dilution.

### Western blot analysis

Western blot was performed to assess protein expression knockdown. Briefly, PfMnmA_cKD parasites were washed then divided and grown with or without 500 nM aTc for 96 h before collecting. NF54 parental parasites were run in parallel. Infected RBCs were washed once with PBS and underwent lysis with cold 0.1% saponin supplemented with cOmplete protease inhibitor (Roche) and 1% phenylmethyl sulfonyl fluoride. RBC lysis was performed on ice for 15 min and parasites were pelleted by centrifugation at 4 °C. Pellets were then washed twice in cold PBS + cOmplete protease inhibitor + phenylmethyl sulfonyl fluoride at 4 °C. Parasite pellets were resuspended in 3× sodium dodecyl-sulfate loading buffer, boiled at 100 °C for 5 min and 1 μl Tris base pH 8 was added. Samples were separated by SDS–polyacrylamide gel electrophoresis using a 4–20% gradient gel (Mini-PROTEAN TGX Precast Gel, 4–20%, Bio-Rad) in Tris–glycine–SDS buffer (Bio-Rad) and transferred to a 0.45 μm nitrocellulose membrane (Bio-Rad). Membranes were blocked in Tris-buffered saline + 0.1% Tween 20 (TBST) + 2% bovine serum albumin overnight, then probed with mouse anti-HA 1:1,000 (BioLegend, Clone 16B12, mouse, 901515) overnight at 4 °C. Membranes were washed in TBST, then incubated overnight with anti-mouse HRP secondary antibody 1:5,000 (Cytiva NA931-1mL) overnight. Membranes (Licor) were then washed in TBST and imaged on a Licor Odyssey platform.

### Growth and morphology assays ±aTc

Growth of the cKD line was assessed ±aTc. PfMnmA_cKD parasites and NF54 parental parasites were grown in 500 nM aTc and sorbitol synchronized as above. On the day of the experiment, aTc was removed by washing three times in aTc-free media. Parasites were then inoculated at 0.15% parasitaemia in 2% RBC in a 96-well plate in triplicate in 500 nM, 3 nM or 0 nM aTc. Cultures were sampled at 72 h, 120 h and 168 h and labelled with SYBR Green I and MitoTracker Deep Red (as DNA and mitochondrial dyes, respectively) and parasitaemias were measured on an iQue Plus flow cytometer. Growth was normalized to the 500 nM aTc samples for each timepoint^93^. Assays were performed in four biological replicates.

To assess morphology, PfMnmA_cKD parasites and NF54 parasites were grown in 500 nM aTc and sorbitol synchronized as above. On the day of the experiment, aTc was removed by washing three times in aTc-free medium and split into either +aTc cultures or −aTc cultures. Parasites were inoculated at 0.2% ring-stage parasitaemia in 3% RBC. Samples were taken at 72, 96, 120, 144 and 168 h post wash-off for all cultures. On the day of sampling, thin smears were stained with Giemsa stain. Next, 200–300 total RBC were counted per condition. The per cent of healthy appearing ring, trophozoite or schizont parasites were counted and microscopy images were taken for all stages at each timepoint (Fig. 4c, Extended Data Figs. 5d and6 and Source data). Total parasitaemia and individual-stage parasitaemias were calculated and graphed using Graphpad Prism. For recovery assays, PfMnmA_ckd parasites that were grown for 168 h with and without aTc were washed three times in aTc-free medium and then again inoculated at 0.2% parasitaemia into medium ±aTc. Sampling was performed as above at 72, 96, 120, 144, 168 and 240 h post wash (Supplementary Fig. 1).

### LC–MS/MS identification of modified ribonucleosides in tRNA PfMnmA_cKD ±aTc

Modified ribonucleosides, specifically mcm^5^s^2^U, m^6^A and m^22^G, were assessed in PfMnmA_cKD parasites cultured with or without aTc. Parasites were sorbitol synchronized and assays started at the trophozoite stage (for the 0, 48 and 96 h timepoints). A separate ring-stage culture was collected at 72 h to generate an additional trophozoite sample. A subset of parasites was collected at the start of the experiment to produce the *t* = 0 sample. For the remainder, aTc was removed by washing three times in aTc-free medium and parasites were then split into 0 nM aTc or 500 nM aTc cultures. Parasites were collected ±aTc at 48, 72 and 96 h timepoints by saponin lysis, as above. Purified *P.* *falciparum* tRNAs from two biological replicates of selected timepoints were hydrolysed enzymatically as described previously^62^. A Waters Acuity BEH C18 column (50 × 2.1 mm inner diameter and 1.7 µm particle size) was used to resolve the digested ribonucleosides in a two-buffer eluent system, with buffer A consisting of water with 0.02% (vol/vol) formic acid and buffer B consisting of acetonitrile with 0.02% (vol/vol) formic acid. All solvents used were LC–MS grade. HPLC was performed at a flow rate of 300 μl min^−1^. The gradient of acetonitrile with 0.02% (vol/vol) formic acid was as follows: 0–5 min, 0–1%; 5–7 min, 1–3%; 7–9 min, 3–7%; 9–10, 7–10%; 10–12 min, 10–12%; 12–13 min, 12–15%; 13–15 min, 15–20%; 15–16 min, 20–75%; 16–17 min 75–100%; 17–20 min, held at 100%, 20–21 min, 100–0%; and 21–25 min, held at 0%. The HPLC column was directly connected to an Agilent 6495 triple quadrupole mass spectrometer with electrospray ionization Jetstream operated in positive ion mode. The voltages and source gas parameters were as follows: gas temperature 200 °C, gas flow 11 l min^−1^, nebulizer 20 psi, sheath gas temperature 300 °C, sheath gas flow 12 l min^−1^, capillary voltage 3,000 V and nozzle voltage 0 V. The multiple reaction monitoring mode was used to detect product ions derived from the precursor ions for all the RNA modifications. Instrument parameters, including the collision energy, were optimized to maximize the sensitivity of detecting modifications. Signal intensities for each ribonucleoside were normalized by dividing them by the sum of the UV signal intensities of the four canonical ribonucleosides as recorded with an in-line UV spectrophotometer at 260 nm.

### RSAs

Parasites were synchronized using magnetic column purification followed by sorbitol synchronization, as described above. RSAs were conducted as previously described, with minor adaptations for cKD kinetics^7^. Briefly, NF54 and PfMnmA_ckd parasites were washed 96 h before beginning the experiment then split into media containing 30 nM aTc and 0 nM aTc. On the day of the experiment, tightly synchronized 0–6 hpi rings were exposed to two-point dilutions of DHA starting at 700 nM to 0 nM for 6 h at 1% parasitaemia and 2% haematocrit, washed three times with RPMI medium to remove the drug and transferred to fresh 96-well plates that contained 30 nM, 3 nM or 0 nM aTc to assess knockdown post-drug exposure (Fig. 4d). A 5,000 nM DHA well was run as a kill control for background gating. Parasites were incubated for 72 h in drug-free medium at the indicated aTc concentrations above. Removal of media and resuspension of parasite cultures was performed on a Freedom Evo 100 liquid-handling instrument (Tecan). Parasitaemias were measured at 72 h by flow cytometry as noted above. Parasite survival was expressed as the percentage value of the parasitaemia in DHA-treated samples divided by the parasitaemia in no-drug samples processed in parallel. Statistical significance was determined using non-parametric, two-tailed Mann–Whitney *U*-tests (GraphPad Prism 9 software). Raw data and statistics are listed in Source data, Extended Data Fig. 8 and Supplementary Fig. 2.

### Drug assays

Seventy-two-hour drug assays were performed as previously described with minor modifications. As above, NF54 and PfMnmA_ckd parasites were washed 96 h before beginning the experiment, then split into media containing 30 nM aTc or 0 nM aTc. Asynchronous, ABS parasites were plated at 0.3–0.7% parasitaemia and 1% haematocrit in 96-well plates and incubated with a ten-point, twofold range of drug concentrations with either 30 nM aTc or 0 nM aTc (Extended Data Fig. 9a). Plates were incubated at 37 °C for 72 h and parasitaemias were measured by flow cytometry. IC_50_ values were calculated by non-linear regression analysis. Statistical significance was determined using Mann–Whitney *U*-tests (Source data).

### Heat shock assays

Heat shock assays were performed as previously described with minor modifications^76^. As above, NF54 and PfMnmA_ckd parasites were grown with aTc. Parasites were synchronized using magnetic column purification, followed by sorbitol synchronization as described above. At 96 h before beginning the experiment, cultures were washed then split into media containing 30 nM aTc or 0 nM aTc. When synchronous parasites reached the mature trophozoite and early schizont stages (26–35 hpi), they were plated at 1% parasitaemia and 2% haematocrit in 6-well plates. One set of plates containing all parasite lines was incubated at 41.5 °C for 3 or 6 h, then returned to 37 °C for the remainder of the parasites’ intra-erythrocytic life cycle. The control set of plates were incubated at 37 °C in parallel. Parasitaemia was measured by flow cytometry after merozoite reinvasion in six technical replicates per line. Survival percentages were determined by the ratio of each heat-shocked line’s average parasitaemia against the average parasitaemia of the corresponding control line (Source data).

### Reporting summary

Further information on research design is available in the Nature Portfolio Reporting Summary linked to this article.

### Supplementary information

Supplementary Information Supplementary results, discussion, Figs. 1–4, figure legends, Tables 1–10 and Source data for Supplementary Figs. 1 and 2.

Reporting Summary

### Source data

Source Data Fig. 1 Raw data for tRNA modification levels.

Source Data Fig. 2 TMT labelling scheme, PSMs, MSstats output, raw values for heat map and for Extended Data Fig. 2.

Source Data Fig. 3 Codon counts for all *P.* *falciparum* proteins, codon counts for top up- and downregulated proteins, codon usage versus transcriptomic changes for up- and downregulated proteins with respect to Lys, His and Asp.

Source Data Fig. 4 Raw data for cKD growth assays, morphology and RSA data for *P.* *falciparum* cultured without aTc before ART pulse.

Source Data Fig. 5 Raw data for IC_50_ and IC_90_ values as determined by 72 drug assays for anti-malarials, raw data for heat shock survival assay.

Source Data Extended Data Fig. 3 Codon use of His and Asp compared with transcriptomics in the up- and downregulated proteins in Dd2R539T post-DHA pulse.

Source Data Extended Data Fig. 4 K13 protein levels from LC–MS/MS proteomics data.

Source Data Extended Data Fig. 5 Uncropped ethidium bromide gels and western blot membranes. For growth assays, source data are in the file for Fig. 4.

Source Data Extended Data Fig. 7 Raw values for tRNA modification analysis in PfMnmA_cKD parasite line ±aTc.

Source Data Extended Data Fig. 8 Raw data RSA data for *P.* *falciparum* cultured with aTc before ART pulse.

Source Data Extended Data Fig. 9 Raw data for *P.* *falciparum* heat shock assay. Seventy-two-hour drug assay data can be found in source data for Fig. 5.

## Data Availability

TMT-tagged proteomics data are available through the PRIDE repository accession PXD043747 (DOI: 10.6019/PDX043747, username: reviewer_pxd043747@ebi.ac.uk, password: jZKeFyVs). Previously published transcriptomics data are available in the National Center for Biotechnology Informatio’s Gene Expression Omnibus with the identifier GSE151189 (ref. ^23^). *P.* *falciparum* GO analyses and localization and gene essentiality predications were obtained from PlasmoDB Release 63. All other data supporting the findings of this study are available within the paper, its Source data and its Supplementary Information. Source data are provided with this paper.
