# Supplementary material for: tRNA modification reprogramming contributes to artemisinin resistance in Plasmodium falciparum
Source: Nat Microbiol. 2024 Apr 17;9(6):1483–98. doi: 10.1038/s41564-024-01664-3 (PMC11153160; doi:10.1038/s41564-024-01664-3)
Supplement: Supplementary file 1 — Supplementary results, discussion, Figs. 1–4, figure legends, Tables 1–10 and Source data for Supplementary Figs. 1 and 2. [file 41564_2024_1664_MOESM1_ESM.pdf]

nature microbiology

Article https://doi.org/10.1038/s41564-024-01664-3

# tRNA modification reprogramming contributes to artemisinin resistance in *Plasmodium falciparum*

In the format provided by the authors and unedited

# Supplementary Information

## tRNA modification reprogramming contributes to artemisinin resistance in *Plasmodium falciparum*

**Jennifer L. Small-Saunders, Ameya Sinha, Talia S. Bloxham, Laura M. Hagenah, Guangxin Sun, Peter Preiser, Peter Dedon, David A. Fidock**

### Supplementary Results, Discussion, Figures and Tables

**Supplementary Results**……………………………………………………………...……page 3

**Supplementary Discussion**……………………..……………………………………..……page 7

**Supplementary References**……………………..…………………………………..……..page 9

**Supplementary Figure 1:** PfMnmA parasites grown persistently without aTc can persist as ring stage parasites, while those regrown on aTc can restore normal growth…………………....page 11

**Supplementary Figure 2: :** Control ring stage survival assay demonstrating that knockdown of PfMnmA has no effect on survival after chloroquine (CQ) exposure………..…………...page 12

**Supplementary Figure 3:** $IC_{90}$ data shown as mean +/- SEM from 72-hr dose response assays of asynchronous NF54 and PfMnmA knockdown parasites………………………………....page 13

**Supplementary Figure 4:** Flow cytometry gating strategy……………………………….....page 14

**Supplementary Figure Legends.**.……………………………... ……...........................page 15

**Supplementary Table 1.** Differentially altered proteins in Dd2 or $Dd2^{R539T}$ parasites after DHA or DMSO exposure..…………………..……………………………………..…………...page 16

**Supplementary Table 2.** Codon use and transcriptomic change data summary for up- and down-regulated proteins in $Dd2^{R539T}$ after DHA pulse……………………………………......page 17

**Supplementary Table 3.** Increased proteins in $Dd2^{R539T}$ parasites post-DHA pulse regulated by Lys codon bias translation...........................................................................................page 18

**Supplementary Table 4.** Decreased proteins in $Dd2^{R539T}$ parasites post-DHA pulse regulated by Lys codon bias translation……………………………………………………………page 19

**Supplementary Table 5.** Increased proteins in $Dd2^{R539T}$ parasites post-DHA pulse regulated by His codon bias translation…………………………………………………………………page 20

**Supplementary Table 6.** Decreased proteins in $Dd2^{R539T}$ parasites post-DHA pulse regulated by His codon bias translation..………………………………......................................page 21

**Supplementary Table 7.** Increased proteins in Dd2$^{R539T}$ parasites post-DHA pulse regulated by Asp codon bias translation……………………………………………………………………page 22
**Supplementary Table 8.** Decreased proteins in Dd2$^{R539T}$ parasites post-DHA pulse regulated by Asp codon bias translation……………………………………………………...…………page 23
**Supplementary Table 9.** Codon use bias between up and down regulated proteins in Dd2$^{R539T}$ parasites post DHA pulse likely regulated by Lys, His and/or Asp codon bias translation………………………………………………………………………………………page 24
**Supplementary Table 10.** List of oligonucleotides used in this study.………....................page 25
**Source Data Supplementary Figure 1.** Raw data for morphology analysis of PfMnmA conditional knockdown grown with or without aTc for 168 hours then regrown with and without aTc…………………………………………………………………………………………........page 26
**Source Data Supplementary Figure 2.** Ring stage survival assay values of PfMnmA_cKD parasite lines grown without aTc prior to chloroquine drug pulse..……………………...........page 27

## Supplementary Results

### ART-R parasites alter their proteome after DHA exposure

We compared differentially regulated proteins at t=0 vs 12 hr post-DMSO for Dd2 and Dd2$^{R539T}$ parasites. For the t=0 hr Dd2 sample, 88 proteins were enriched compared to the 12 hr timepoint. Of these, 25 corresponded to proteins with a gene ontology (GO) enrichment category of host cell entry, which was also enriched in the Dd2$^{R539T}$ t=0 hr sample (**Fig. 2b; Supplementary Table 1**). 143 proteins were upregulated at the 12 hr time point compared to t=0 in DMSO-treated Dd2 parasites. 95 of these proteins, corresponding to ATP synthase subunits and other ion transmembrane transport proteins, did not change in t=12 hr DMSO-treated Dd2$^{R539T}$. 48 of these 143, however, were also upregulated in DMSO-treated Dd2$^{R539T}$ parasites at t=12 hr. These included mitochondrial proteins and the export translocator PTEX-complex. Proteins involved in response to unfolded proteins were significantly upregulated in Dd2$^{R539T}$ parasites at 12 hr post-DMSO, but not in Dd2 parasites (**Fig. 2c; Supplementary Table 1**).

We next compared differentially regulated proteins in Dd2 vs Dd2$^{R539T}$ parasites after ART exposure vs DMSO exposure. In Dd2 parasites, 42 proteins were upregulated (**Fig. 2d**) and 36 proteins were downregulated in ART vs DMSO conditions (**Fig. 2e**), which corresponded to chromatin organizing proteins and metabolic glycolysis pathway proteins respectively. Dd2$^{R539T}$ had a greater number of proteins changing post ART exposure vs DMSO exposure, with 139 upregulated (**Fig. 2d**) and 149 downregulated (**Fig. 2e**) post ART-treatment. Surprisingly, proteins involved in entry into the host were upregulated and those involved in translation (58 genes) were strongly downregulated (**Supplementary Table 1**). This is consistent with translational arrest of the Dd2$^{R539T}$ parasites after ART but not DMSO exposure[1].

Lastly, we screened for proteins differentially regulated in DHA-treated Dd2$^{R539T}$ parasites that did not significantly change in DHA-treated Dd2 parasites to identify the selective response of DHA-treated mutant parasites. This analysis revealed that 44 proteins were significantly upregulated and were involved in protein refolding and mitochondrial physiology. Of the 70 downregulated genes, several were involved in translation, with 14 genes in the ribosomal biogenesis pathway (**Supplementary Table 1**). This again supports translational arrest of Dd2$^{R539T}$, but not Dd2, parasites after ART exposure.

### Pf3D7_1019800 (PfMnmA) knockout confirmation

Gene editing of the NF54_PfMnmA_cKD line (referred to below as PfMnmA_cKD) was confirmed using PCR (**Extended Data Fig. 5a**) and Sanger sequencing. Western blot detection of the C-terminal 3×HA

tag appended to the cKD regulated protein demonstrated loss of the expected 134 kDa band in samples cultured without aTc for 96 hr. While detection of this low-abundance protein was difficult, the reproducibility of the result indicated that we were likely achieving a significant knockdown (**Extended Data Fig. 5b**).

### Pf3D7_1019800 (PfMnmA) is required for parasite development

To assess the impact of PfMnmA knockdown on parasite growth and morphology, NF54 and PfMnmA_cKD parasites were inoculated into media containing 0 nM, 3 nM (low) and 500 nM (high) aTc. PfMnmA_cKD cultured in low or no aTc displayed a slow onset of parasite death as compared to parasites cultured with high aTc, while NF54 parasites had no change in growth (**Fig. 4b; Extended Data Fig. 5C**). In the no aTc sample, parasitemia began to decrease at 72 hr and progressed to 61% and 22% of the high aTc control growth measured at 120 hr and 168 hr, respectively (**Fig. 4b**). In contrast, there was no change in growth of NF54 controls, irrespective of the aTc concentration (**Extended Data Fig. 5c**). This progressive onset of parasite death correlated well with the other PfMnmA cKD study[2]. We suspect that our cKD likely has low level MnmA translation secondary to incomplete translational repression and leaky regulation, which would explain the slow onset of parasite death[2,3]. Nonetheless, this delay made it possible to phenotype our PfMnmA_cKD line.

At 72 hr after aTc removal, we observed some healthy rings, trophozoites and schizonts, despite the slowdown in parasite growth. By 120 hr after aTc removal there were evident defects in schizont morphology, suggesting a role for this protein in schizont development (**Fig. 4c; Extended Data Figs. 5d, 6a**). Further studies showed that schizogony could be restored by adding back aTc (**Supplementary Fig. 1).** Intriguingly, the cKD parasites initially cultured without aTc and continued without aTc persisted as low-level ring stage parasites, although it is unclear if these were new or quiescent rings. Given the importance of the ring stage and its extended duration of development in ART resistance[1], this observation suggests that PfMnmA loss may also have a similar effect on ring stage development or quiescence.

Finally, we used LC-MS/MS to assess global $mcm^5s^2U$ modification levels in PfMnmA_cKD parasites ± aTc. PfMnmA_cKD parasites were washed and inoculated into media containing 0 nM or 500 nM aTc and harvested for tRNA analysis at 0 hr and 48, 72 and 96 hr post aTc removal. Control parasites were cultured with aTc for the duration of the experiment. PfMnmA_cKD parasites cultured without aTc (-aTc) had lower levels of $mcm^5s^2U$, as compared to the same parasites cultured with aTc (+aTc; **Extended Data Fig. 7a**). This trend was consistent in both biological replicates (**Extended Data Fig. 7d**). Total levels of the $mcm^5s^2U$ modification in -aTc parasites were significantly reduced, as compared to the

+aTc controls ($p<0.05$). In contrast, there were no significant changes in levels of either the $m^{2,2}G$ or $m^6A$ modifications between - and + aTc cultures in the PfMnmA_cKD line (**Extended Data Fig. 7b,c,e,f**). These findings suggest that the PfMnmA knockdown led to specific decreases in global $mcm^5s^2U$ modification levels, although this did not abolish the modification fully, likely because of the contribution of the cytosolic $s^2U$ biosynthetic pathway.

## Knockdown of MnmA results in increased resistance to artemisinin

PfMnmA_cKD parasites that underwent protein knockdown (no aTc for 96 hr) prior to DHA exposure demonstrated an aTc concentration-dependent increase in ART survival, as compared to NF54 (**Fig. 4e**). At 700 nM DHA, NF54 parasites did not survive (0.3% to 1.6% survival rates). In contrast, PfMnmA_cKD parasites cultured with 30, 3 or 0 nM aTc post DHA pulse had 2.2%, 3.9% and 5.1% survival, respectively, which was significantly more than their NF54 controls ($p<0.05\%$). At 350 nM DHA, differences were more pronounced. NF54 parasites had little to no survival (1.7% to 2.5% irrespective of the aTc concentration). Knockdown parasites had significantly greater survival rates, with 5.2%, 6.7% and 11% measured at 30 nM, 3 nM and 0 nM aTc, respectively. This aTc concentration-dependent increased survival of the PfMnmA_cKD parasites was evident at DHA concentrations as low as 2.7 nM (**Extended Data Fig. 8a**).

## MnmA knockdown parasites show altered antimalarial susceptibility

Azithromycin and fosmidomycin act on the apicoplast by inhibiting the apicoplast ribosome or the DOXP reductoisomerase enzyme in the non-mevalonate pathway, respectively[4,5]. Knockdown of MnmA led to low-level but significant two-fold sensitization to both drugs, as compared to non-knockdown conditions (**Fig. 5b,c**). aTc had no effect on $IC_{50}$ values for NF54. These data are consistent with prior localization of PfMnmA to the parasite apicoplast and its requirement for maintenance of this organelle[2]. No change was observed for the mitochondrial DHODH inhibitor DSM265 (**Fig. 5d**). In contrast, knockdown led to a 1.8-fold increase in the $IC_{50}$ of the cytochrome bc1 inhibitor, atovaquone (increasing from a mean of 0.5 nM to 0.9 nM for “translation on” vs “translation off” parasites; **Fig. 5e**). Interestingly, knockdown parasites never had more than 90% killing even at high μM concentrations, which suggests a small population of atovaquone-tolerant parasites (**Fig. 5e,f**). This concentration-response profile differed from atovaquone-resistant control parasites that harbored a CYTb V259L mutation and that showed >100-fold increases in both the $IC_{50}$ and $IC_{90}$ values relative to the Dd2 parent (**Fig. 5f**).

*P. falciparum* response to ART derivatives has been linked to the ubiquitin/proteasome system, the unfolded protein response, and heat shock, and is related to ART-induced proteotoxic stress[6-10]. We therefore tested our knockdown against proteasome inhibitors and elevated temperatures. Knockdown

parasites in 0 nM aTc showed a small but significant increase in their mean $IC_{50}$ value with the proteasome inhibitor WLL, as compared to non-knockdown conditions (**Fig. 5g**). This suggested that $s^2U$ hypomodification may also play a protective role in response to proteotoxic stress, as well as ART stress. No differences were noted in PfMnmA_cKD parasites after aTc removal in response to piperaquine, pyronaridine or lumefantrine (**Extended Data 9b-d**). Both knockdown and NF54 parasites showed a slight sensitization to mefloquine (**Extended Data Fig. 9e**). Interestingly, lumefantrine alone demonstrated a shift at the $IC_{90}$ level (**Supplementary Fig. 2a-h**). Finally, we hypothesized that changes in PfMnmA levels may also affect heat sensitivity, given their decreased susceptibility to DHA and WLL. We prepared highly synchronized MnmA_cKD and NF54 trophozoites that were cultured at 0 nM aTc for 96 hr prior to being exposed to 42˚C for 3 or 6 hr[9]. No changes in survival were seen in the knockdown parasites in response to heat shock (**Extended Data Fig. 9f**). Our data highlight that despite overlaps between ART resistance, proteotoxic stress and the heat shock response, the underlying mechanisms are more nuanced.

## Supplementary Discussion

Here, we provide compelling evidence that drug-resistant parasites have adapted the tRNA modification reprogramming regulatory mechanism to differentially respond to ART stress and aid in survival. Drug-sensitive parasites increased their levels of $mcm^5s^2U$, $mcm^5Um$ or $m^{6,6}A$ modifications in response to DHA. In contrast, drug-resistant parasites had extensive tRNA hypomodification post DHA treatment, specifically decreasing $mcm^5s^2U$ and mCm, as compared to isogenic ART-S parasites, after DHA but not control treatment. Changes were observed in early ring-stages (6 hr post initiation of DHA treatment), before $Dd2^{R539T}$ and Dd2 diverged in their ring-stage developmental progression[1]. These data suggest a dynamic reprogramming of tRNA modifications, specifically $mcm^5s^2U$, in ART-R parasites as they respond to drug-induced stress. Interestingly, an analogous situation exists in melanoma tumor cells, whereby alterations in tRNA modifications can contribute to chemotherapy resistance[11]. Indeed, changes in tRNA modifications have increasingly been implicated in the pathogenesis of other diseases including human mitochondrial disorders and *Mycobacterium tuberculosis* infection[12-14].

A second but not mutually exclusive hypothesis on how $s^2U$ hypomodification may lead to resistance is that this hypomodification can enhance the parasite unfolded protein response[1,7,8]. In *S. cerevisiae,* thiolation pathway mutants have increased amounts of unfolded proteins. This leads to chronic proteotoxic stress and affords survival advantages in response to specific stressors, such as the ER stressor tunamycin[15]. In *P. falciparum*, decreased $s^2U$ modifications may prime a subpopulation to have a chronic level of proteotoxic stress, thereby allowing for a more rapid unfolded protein response post DHA exposure (**Extended Data Fig. 10b**).

Here we show that ART-R parasites can employ codon-use bias as a mechanism to regulate mRNA translation and the proteome in response to ART-induced stress. The 12 hr proteome (i.e. post 6-hr DHA pulse and recovery) of the $Dd2^{R539T}$ parasites displayed a significant bias for Lys, Asp and His codons. Upregulated proteins were enriched for $Lys^{AAA}$, $Asp^{GAT}$, and $His^{CAT}$ and downregulated proteins were enriched for the cognate codons ($Lys^{AAG}$, $Asp^{GAC}$, $His^{CAC}$). This Lys codon bias further linked our $mcm^5s^2U$ tRNA modification changes and our proteomic data. Interestingly, $Asp^{GAT/GAC}$ and $His^{CAT/CAC}$ are regulated by queuosine modifications[16], which link translation with cellular nutritional status[16,17]. Although we were unable to detect this modification, our observation of Asp and His codon-use biases provides an avenue for future study. Usage of all codons, tabulated for each of the 5,363 predicted proteins in the Pf proteome provides a rich resource to examine codon bias across the entire proteome (**Source Data Fig. 3**).

The Lys codon usage pattern we observed in ART-R, $s^2U$ hypomodified parasites was unexpected. In *S. cerevisiae* deficient for thiolation pathway modifiers, ribosomes accumulate at GAA, CAA and AAA codons, leading to slower translation and downregulation of protein synthesis mediators. Ribosome pausing on -A ending codons then leads to destruction of AAA enriched transcripts and enrichment of proteins with $Lys^{AAG}$ codons[18]. Yet, we observed enrichment of $Lys^{AAA}$ in upregulated proteins. It is unlikely that the convergence of $mcm^5s^2U$ modification changes, transcriptomic changes in $s^2U$ biosynthetic pathway enzymes, and proteomic Lys codon bias, is coincidental. The observed $Lys^{AAA}$ enrichment may instead relate to temporal differences in our proteomics and tRNA modification analyses (**Extended Data Fig. 10b).** Future studies will be necessary to fully elucidate the kinetics of tRNA modifications and their effects on the parasite proteome.

We initially targeted the MnmA enzyme, despite its putative organellar localization, for three reasons: 1) Transcriptomic data demonstrated that the MnmA pathway was differentially regulated in $Dd2^{R539T}$ parasites, as compared to Dd2 parasites, post DHA exposure[1]; 2) this thiouridine synthetase is differentially regulated in heat shock-sensitive *P. falciparum* mutants, as compared to NF54 controls, after heat shock[10]; and 3) the PfMnmA gene was predicted to be essential, whereas the *P. falciparum* Ncs6 ortholog (Pf3D7_0612600) was not[3]. Our data show PfMnmA is essential for parasite growth and schizont development. A separate study that created a PfMnmA conditional knockdown also demonstrated essentiality and showed that *P. falciparum* likely has a truncated, bacterial-like $s^2U$ biosynthetic pathway[2]. MnmA was also shown to be important in apicoplast maintenance in both *P. falciparum* and *T. gondii*[2,19].

Unexpectedly, the PfMnmA knockdown led to decreased susceptibility to atovaquone, a cytochrome bc1 inhibitor that is known to retain activity against quiescent parasites[20]. Our observed resistance appears distinct from cytochrome bc1 inhibition, as an atovaquone-resistant CYTB1 V259L mutant line showed complete killing at high atovaquone concentrations. In human and yeast mitochondria, loss of the MnmA homolog, TRMU, leads to decreased mitochondrial respiration[21]. An analogous situation with *P. falciparum* may explain why our knockdown of PfMnmA (localized to the apicoplast[2]) is tolerant to atovaquone. Given the proximity of the apicoplast and the mitochondria in *P. falciparum*, it will be interesting to test whether PfMnmA may also serve as the mitochondrial tRNA thiouridine transferase.

## References


1 Mok, S. *et al.* Artemisinin-resistant K13 mutations rewire *Plasmodium falciparum*'s intra-erythrocytic metabolic program to enhance survival. *Nat Commun* **12**, 530 (2021). https://doi.org:10.1038/s41467-020-20805-w

2 Swift, R. P., Elahi, R., Rajaram, K., Liu, H. B. & Prigge, S. T. The *Plasmodium falciparum* apicoplast cysteine desulfurase provides sulfur for both iron sulfur cluster assembly and tRNA modification. *elife* **12**, e84491 (2023). https://doi.org:10.7554/eLife.84491

3 Zhang, M. *et al.* Uncovering the essential genes of the human malaria parasite *Plasmodium falciparum* by saturation mutagenesis. *Science* **360**, eaap7847 (2018). https://doi.org:10.1126/science.aap7847

4 Lell, B. *et al.* Fosmidomycin, a novel chemotherapeutic agent for malaria. *Antimicrob Agents Chemother* **47**, 735-738 (2003). https://doi.org:10.1128/AAC.47.2.735-738.2003

5 Blasco, B., Leroy, D. & Fidock, D. A. Antimalarial drug resistance: linking *Plasmodium falciparum* parasite biology to the clinic. *Nat Med* **23**, 917-928 (2017). https://doi.org:10.1038/nm.4381

6 Mok, S. *et al.* Population transcriptomics of human malaria parasites reveals the mechanism of artemisinin resistance. *Science* **347**, 431-435 (2015). https://doi.org:10.1126/science.1260403

7 Dogovski, C. *et al.* Targeting the cell stress response of *Plasmodium falciparum* to overcome artemisinin resistance. *PLoS Biol* **13**, e1002132 (2015). https://doi.org:10.1371/journal.pbio.1002132

8 Bridgford, J. L. *et al.* Artemisinin kills malaria parasites by damaging proteins and inhibiting the proteasome. *Nat Commun* **9**, 3801 (2018). https://doi.org:10.1038/s41467-018-06221-1

9 Tinto-Font, E. *et al.* A heat-shock response regulated by the PfAP2-HS transcription factor protects human malaria parasites from febrile temperatures. *Nat Microbiol* **6**, 1163-1174 (2021). https://doi.org:10.1038/s41564-021-00940-w

10 Zhang, M. *et al.* The apicoplast link to fever-survival and artemisinin-resistance in the malaria parasite. *Nat Commun* **12**, 4563 (2021). https://doi.org:10.1038/s41467-021-24814-1

11 Rapino, F. *et al.* Codon-specific translation reprogramming promotes resistance to targeted therapy. *Nature* **558**, 605-609 (2018). https://doi.org:10.1038/s41586-018-0243-7

12 Suzuki, T. The expanding world of tRNA modifications and their disease relevance. *Nat Rev Mol Cell Biol* **22**, 375-392 (2021). https://doi.org:10.1038/s41580-021-00342-0

13 Dedon, P. C. & Begley, T. J. Dysfunctional tRNA reprogramming and codon-biased translation in cancer. *Trends Mol Med* **28**, 964-978 (2022). https://doi.org:10.1016/j.molmed.2022.09.007

14 Tomasi, F. G., Kimura, S., Rubin, E. J. & Waldor, M. K. A tRNA modification in ***Mycobacterium*** *tuberculosis* facilitates optimal intracellular growth. *eLife* **12**, RP87146 (2023). https://doi.org:10.7554/eLife.87146

15 Damon, J. R., Pincus, D. & Ploegh, H. L. tRNA thiolation links translation to stress responses in Saccharomyces cerevisiae. *Mol Biol Cell* **26**, 270-282 (2015). https://doi.org:10.1091/mbc.E14-06-1145

16 Tittle, J. M., Schwark, D. G., Biddle, W., Schmitt, M. A. & Fisk, J. D. Impact of queuosine modification of endogenous *E. coli* tRNAs on sense codon reassignment. *Front Mol Biosci* **9**, 938114 (2022). https://doi.org:10.3389/fmolb.2022.938114

17 Tuorto, F. *et al.* Queuosine-modified tRNAs confer nutritional control of protein translation. *EMBO J* **37**, e99777 (2018). https://doi.org:10.15252/embj.201899777

18 Nedialkova, D. D. & Leidel, S. A. Optimization of codon translation rates via tRNA modifications maintains proteome integrity. *Cell* **161**, 1606-1618 (2015). https://doi.org:10.1016/j.cell.2015.05.022

19 Yang, Y. *et al.* The first apicoplast tRNA thiouridylase plays a vital role in the growth of Toxoplasma gondii. *Front Cell Infect Microbiol* **12**, 947039 (2022). https://doi.org:10.3389/fcimb.2022.947039

20 Reyser, T. *et al.* Identification of compounds active against quiescent artemisinin-resistant *Plasmodium falciparum* parasites via the quiescent-stage survival assay (QSA). *J Antimicrob Chemother* **75**, 2826-2834 (2020). https://doi.org:10.1093/jac/dkaa250

21 Umeda, N. *et al.* Mitochondria-specific RNA-modifying enzymes responsible for the biosynthesis of the wobble base in mitochondrial tRNAs. Implications for the molecular pathogenesis of human mitochondrial diseases. *J Biol Chem* **280**, 1613-1624 (2005). https://doi.org:10.1074/jbc.M409306200

## Supplementary Figure 1

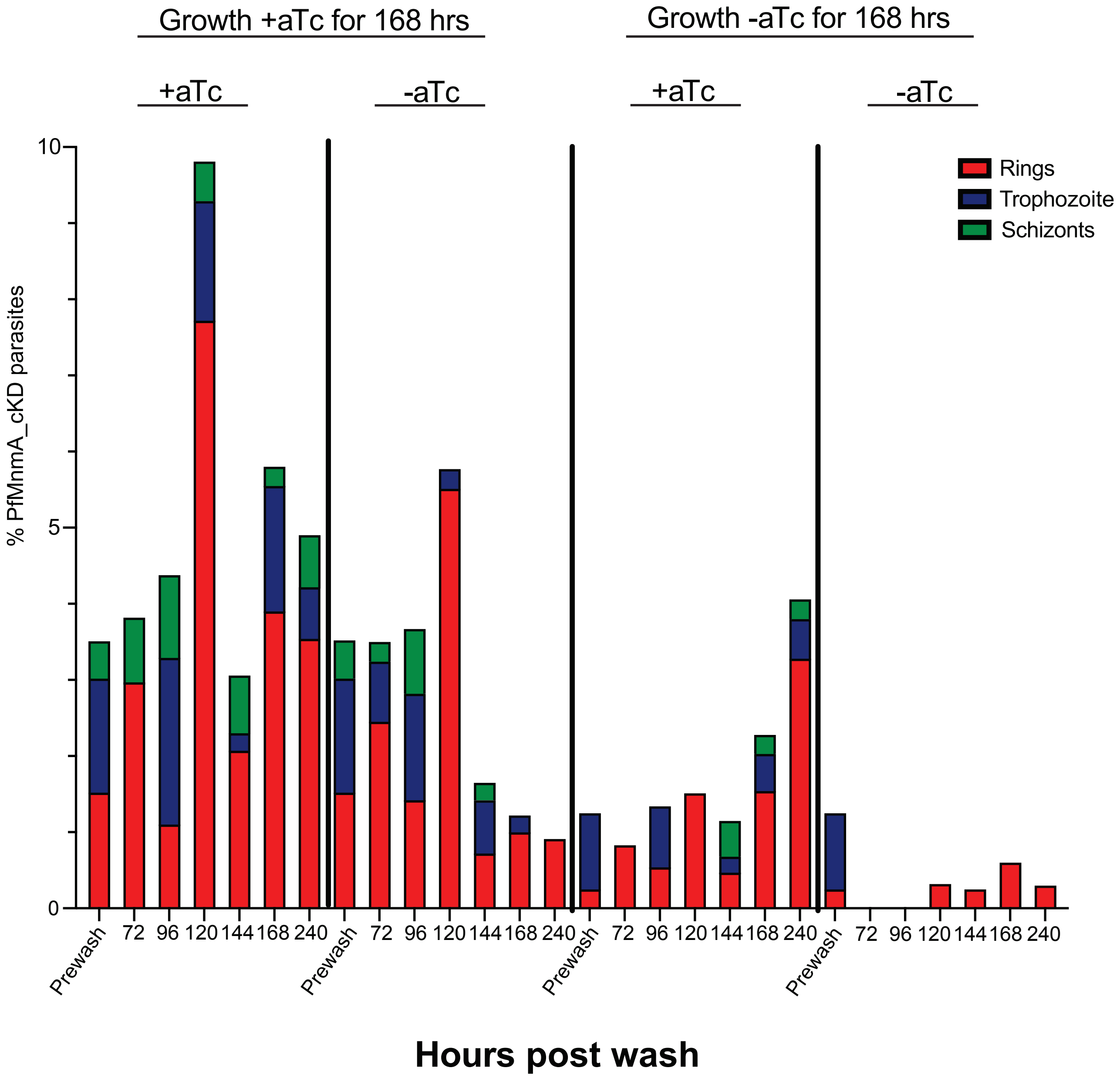

**Supplementary Figure 2**

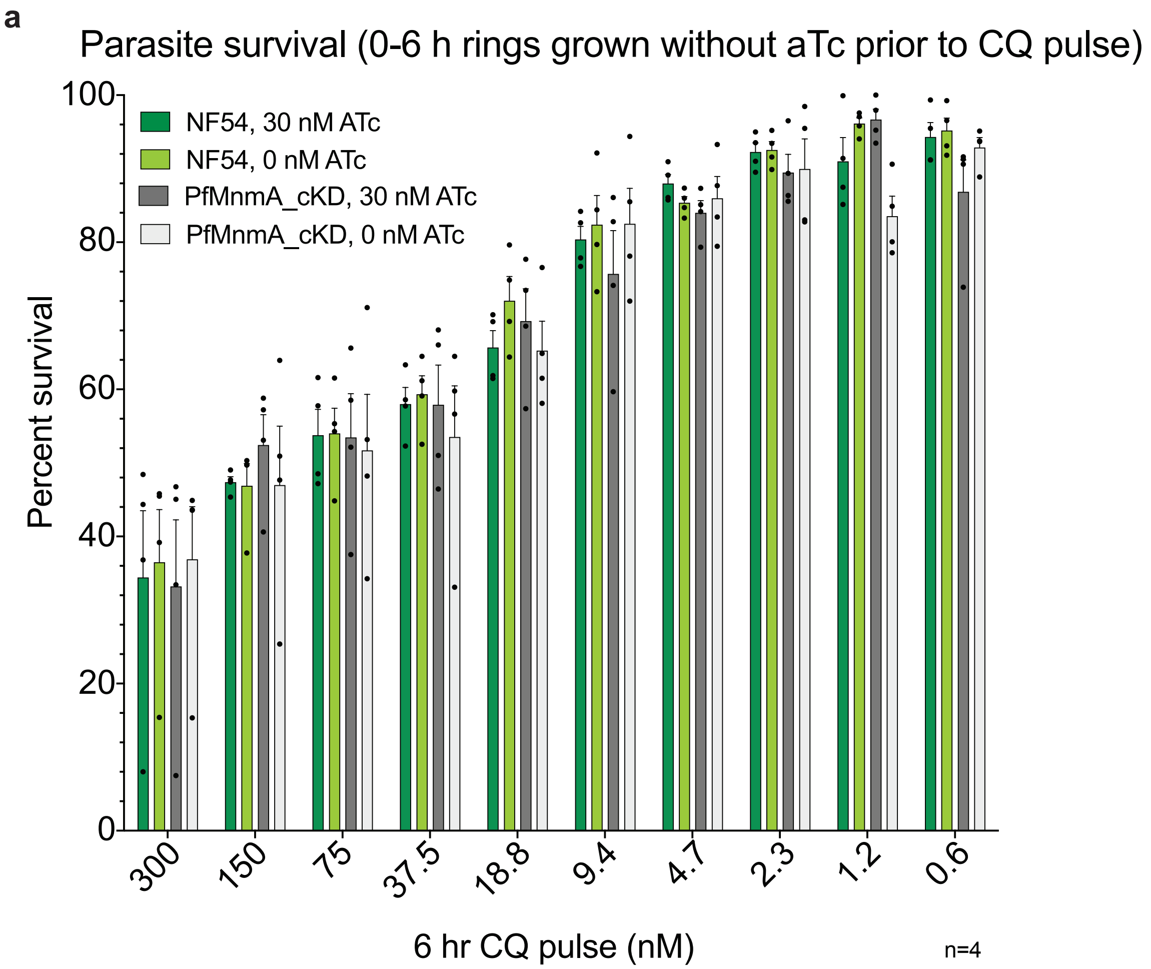


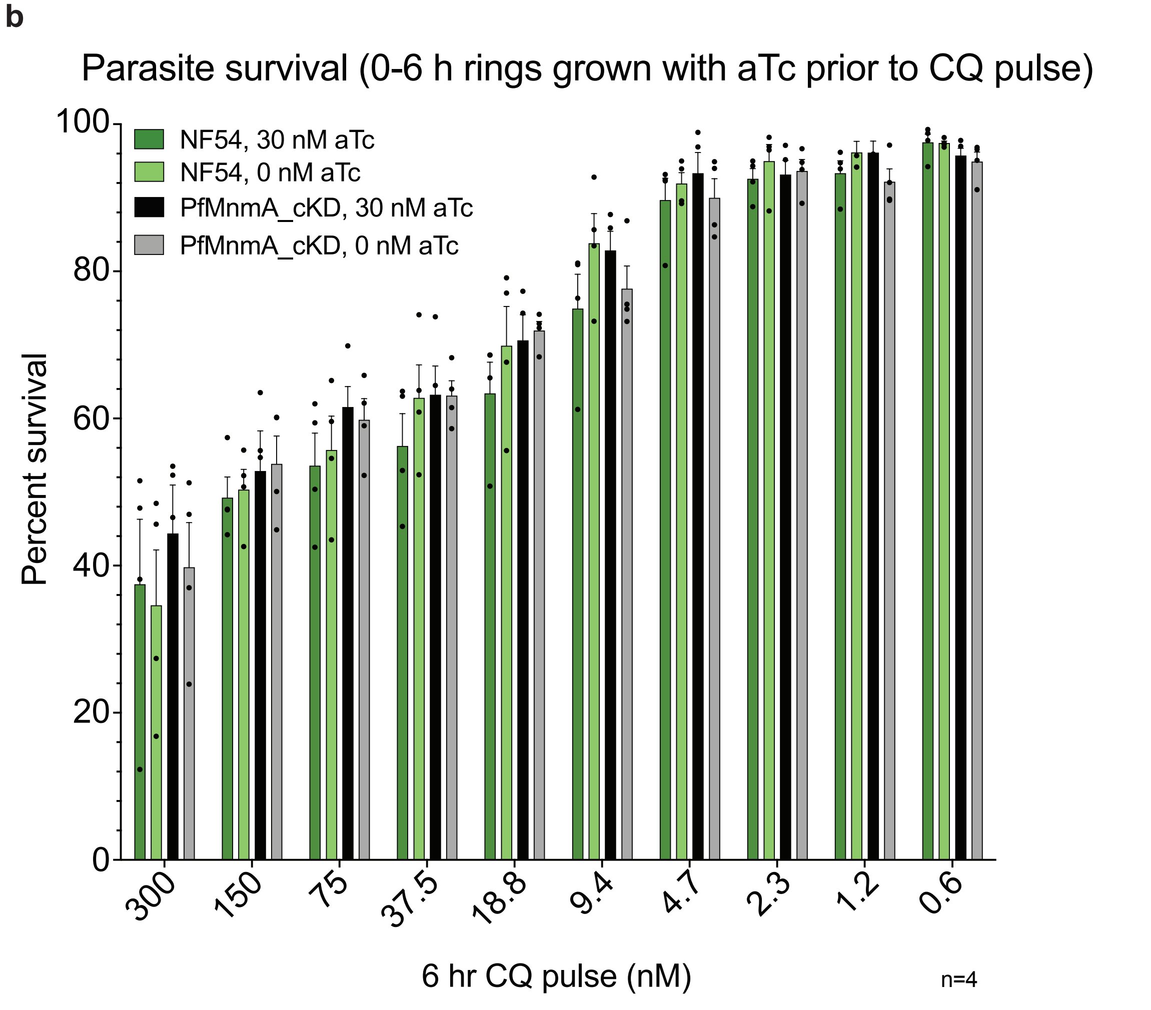

## Supplementary Figure 3

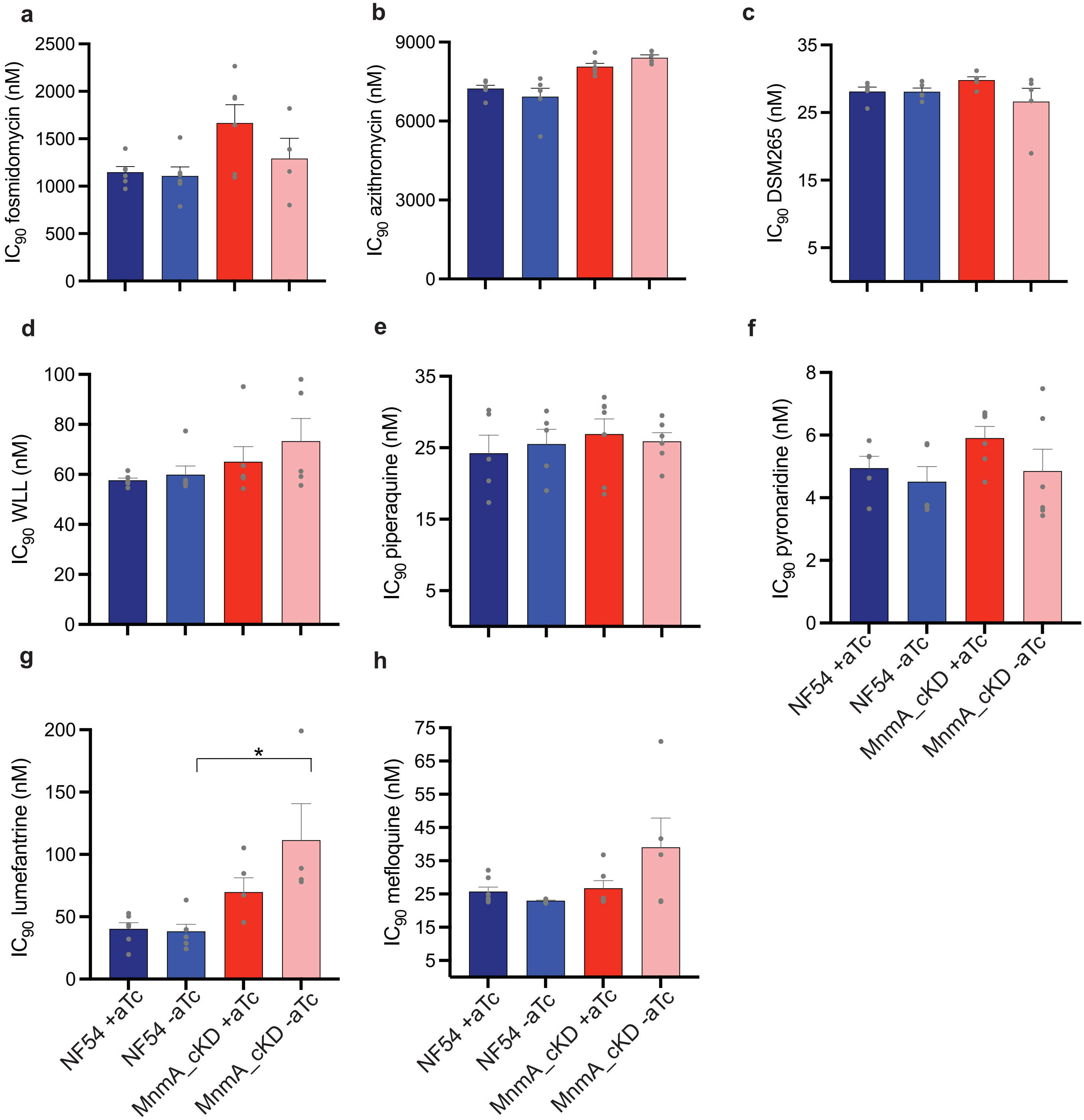

# Supplementary Figure 4

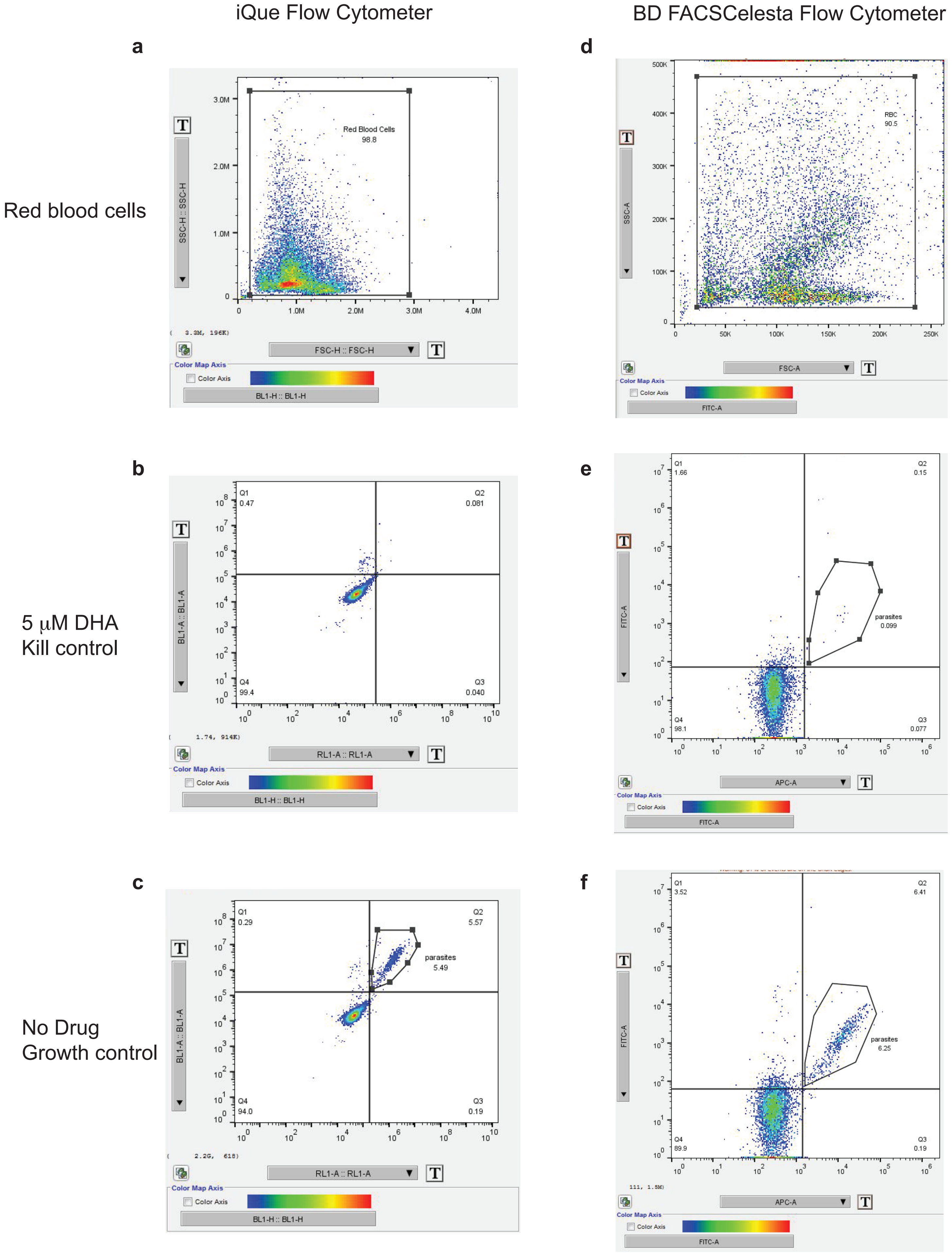

**Supplementary Fig. 1 | PfMnmA parasites cultured continuously without aTc can persist as ring stage parasites, while those regrown on 500 nM aTc can restore normal growth.** PfMnmA parasites were cultured for 168 hr ± aTc (**Fig. 4c**). These parasites were then washed thoroughly, split again into ± aTc and cultured for additional periods ranging from 72 hr to 240 hr. At each of the indicated time points, thin smears were Giemsa stained and 100 RBC were counted (**Source Data Supplementary Fig. 1**). Parasites were divided into ring, trophozoite, and schizont stages with total parasitemia show on the y-axis. For parasites initially cultured with aTc and continued on aTc, there was no growth change. However, parasites that were split into the no aTc group demonstrated loss of schizonts and slowdown of growth. Parasites initially cultured without aTc recovered growth and resumed schizogony within 144 hr of adding aTc, further supporting the hypothesis of incomplete translational repression in our PfMnmA_cKD line.

**Supplementary Fig. 2 | Control ring stage survival assays demonstrating that knockdown of PfMnmA has no effect on survival after chloroquine (CQ) exposure**. RSAs were performed as outlined in **Fig. 4d** but CQ was substituted for DHA. Highly synchronized 0-6 hr ring-stage parasites cultured for 96 hr (**a**) without aTc or (**b**) with aTc prior to drug pulse were exposed to a 6 hr pulse of a range of CQ, drug was washed off, and parasites were allowed to recover on plates with 30 nM aTc or 0 nM aTc for 72 hr. Results demonstrate the percentage of early ring-stage parasites (0-6h hpi) that survived a range of CQ concentrations beginning at 300 nM relative to no-drug control parasites assayed in parallel. Percent survival values are shown as means ± SEM (**Source Data Supplementary Fig. 2**). N=4 independent biological replicates. Statistical significance was determined via two-tailed Mann Whitney *U* tests.

**Supplementary Fig. 3 |** $IC_{90}$ data shown as mean ± SEM (**Source Data Fig. 5**) from 72 hr dose response assays of asynchronous NF54 parasites cultured ± aTc, PfMnmA_cKD parasites cultured with aTc and PfMnmA_cKD parasites cultured without aTc for 96 hr prior to drug pulse for (**a**) fosmidomycin, (**b**) azithromycin, (**c**) DSM265, (**d**) WLL, (**e**) piperaquine, (**f**) pyronaridine, (**g**) lumefantrine, and (**h**) mefloquine. N,n= 5 to 7 independent biological replicates per parasite line. Statistical significance was determined via two tailed Mann Whitney *U* tests. *p<0.05, **p<0.01.

**Supplementary Fig. 4 |** Flow cytometry gating strategy for determining parasitemia. Samples were obtained on either an iQue Flow Cytometer (**a-c**) or a FACS Celesta Flow Cytometer (**e-f**). Flow counts were first gated for red blood cells using FSC and SSC channels (**a,d**). For the iQue, live parasites were determined as positive events for BL1-A and FL1-A (**b,c**). For the FACS Celesta, live parasites were determined as positive events for FITC-A and APC-A (**e,f**). 5 μM dihydroartemisinin (DHA) was used as a kill control to determine background (**b,e**) and no drug controls were used to determine full parasite growth.

**Supplementary Table 1.** Differentially altered proteins in Dd2 and Dd2$^{R539T}$ parasites after DHA or DMSO treatment

| Condition | Line | Number proteins | Notable GO enrichments | Genes |
|---|---|---|---|---|
| 0 h | Dd2 | 88 | | |
| | Dd2$^{R539T}$ | 96 | | |
| | Both | 48 | Microneme | PF3D7_0102500, PF3D7_0323400, PF3D7_0731500, PF3D7_0828800, PF3D7_1028700 |
| | | | Rhoptry | PF3D7_0214900, PF3D7_0423400, PF3D7_0424100, PF3D7_0707300, PF3D7_0722200, PF3D7_0929400, PF3D7_1012200, PF3D7_1017100, PF3D7_1335400 |
| 12 h DMSO | Dd2 | 143 | ATP synthase subunits | PF3D7_0217100, PF3D7_0715500, PF3D7_1024300, PF3D7_1142800, PF3D7_1235700 |
| | | | Ion transmembrane transport proteins | PF3D7_0106300, PF3D7_1211900, PF3D7_1340900, PF3D7_1456800 |
| | Dd2$^{R539T}$ | 83 | Unfolded proteins | PF3D7_1118200, PF3D7_1134000, PF3D7_1215300 |
| | Both | 48 | Mitochondrial proteins | PF3D7_1368600, PF3D7_1124700, PF3D7_0928000, PF3D7_1116800 |
| | | | Export translocator PTEX-complex | PF3D7_1105600, PF3D7_1436300 |
| 12 h DHA vs DMSO | Dd2 | upregulated: 42 | Chromatin organizing proteins | PF3D7_0610400, PF3D7_1308100, PF3D7_1329300 |
| | | downregulated: 36 | Metabolic glycolysis pathway proteins | PF3D7_0610800, PF3D7_1012400, PF3D7_1015900, PF3D7_1023200, PF3D7_1029600, PF3D7_1406300, PF3D7_1436000, PF3D7_1439900, PF3D7_1444800 |
| | Dd2$^{R539T}$ | upregulated: 139 | Entry into the host | |
| | | downregulated: 149 | Translation (58 genes) | |
| 12 hr DHA Dd2$^{R539T}$ (unchanged in Dd2) | Dd2$^{R539T}$ | upregulated: 44 | Protein refolding | PF3D7_0831700, PF3D7_0917900, PF3D7_1232100 |
| | | | Mitochondrial reorganization | PF3D7_1208600, PF3D7_1368600 |
| | | downregulated: 70 | Ribosome biogenesis (14 genes) | |

TMT-tagged proteomics analysis resulted in 1315 proteins with 40,955 peptide spectral matches from Dd2 or Dd2$^{R539T}$ parasites at 0 hr or 12 hr after a 6hr DHA or DMSO pulse. Isogenic, edited Dd2 (BSMUT, artemisinin sensitive) and Dd2$^{R539T}$ (artemisinin resistant) parasites were highly sorbitol s to early ring stages (0-6 hr post invasion, hpi) then pulsed with either 700 nM DHA or 0.1% DMSO. Samples were collected at t=0, parasites underwent thorough drug wash-off at 6 hr and samples were collected at 12 hr. Proteins listed above were from proteomes analyzed at noted conditions for i lines. Gene ontology analysis was performed for subsets of proteins, with notable enrichments and gene IDs noted here. The last row represents the differential response of Dd2R539T parasites after DHA exposure as compared with Dd2$^{R539T}$ parasites sampled at time 0. Proteins that were similar down-regulated in DMSO-treated Dd2$^{R539T}$ parasites and/or Dd2 DHA-treated parasites were excluded, in order to identify protein changes unique to DHA-treated Dd2$^{R539T}$ parasites. Data are represented visually in **Fig. 2b-e**.

**Supplementary Table 2.** Codon use and transcriptomic change data summary for up- and down-regulated proteins in Dd2$^{R539T}$ after DHA pulse.

| | Upregulated Proteins | | | Downregulated Proteins | | |
|---|---|---|---|---|---|---|
| Enriched Codon | LysAAA | HisCAT | AspGAT | LysAAG | HisCAC | AspGAC |
| Cognate Codon | LysAAG | HisCAC | AspGAC | LysAAA | HisCAT | AspCAT |
| Percent of proteins with enriched codon | 47.7 | 31.8 | 31.8 | 34.3 | 32.9 | 21.4 |
| Percent of proteins with cognate codon | 15.9 | 27.3 | 29.5 | 22.9 | 30.0 | 37.1 |
| Percent of proteins without codon bias | 36.4 | 40.9 | 38.6 | 42.9 | 37.1 | 41.4 |
| Percent of transcript changing in opposite direction as protein | 50.0 | 50.0 | 50.0 | 42.9 | 42.9 | 42.9 |
| Percent of codon bias proteins and differentially translated proteins | 27.3 | 13.6 | 18.2 | 14.3 | 11.4 | 7.1 |
| Percent of LysAAA proteins without transcriptional changes | 57.1 | 42.9 | 57.1 | 41.7 | 34.8 | 33.3 |

Codon usage characteristics of up and down-regulated proteins in Dd2$^{R539T}$ after DHA pulse. Proteins identified during proteomic analysis (**Fig 2, Source Data Fig. 2**) that were signficantly increased or decreased ($p<0.05$) in the Dd2$^{R539T}$ parasites 12hr post initiation of DHA exposure. This curated list arose from removal of proteins that also increased in these parasites after DMSO exposure. Codon usage for Lys, His and Asp for each protein was determined using a codon counting algorithm (**Source Data Fig. 3**). Cutoff for codon bias was Z scores >0.5. Transcriptomic data were analyzed to determine fold change values of Dd2$^{R539T}$ parasites from time 0 versus a 6 hr DHA pulse. Values are log2 transformed. Candidates for codon bias translation are proteins shown in gray, which were ones that had codon bias and were increased translationally with decreased or no change in transcription or had codon bias and were decreased translationally with no or increased transcription (**Fig 3c, Extended Data Fig. 4b,c and Source Data for Figs. 3 and Extended Data Fig. 3**).

**Supplementary Table 3.** Increased proteins in $Dd2^{R539T}$ parasites post-DHA pulse regulated by Lys codon bias translation.

| Number | Gene ID | Gene Name | Log2 Protein Fold Change | LysAAA Z score | LysAAG Z score | Log2 RNA Fold Change | Top associated GO terms | GO Localization | Essential |
|---|---|---|---|---|---|---|---|---|---|
| 1 | PF3D7_0625400 | Uncharacterized protein | 0.63 | 0.97 | -0.97 | -0.26 | N/A | N/A | Yes |
| 2 | PF3D7_0321100 | Uncharacterized protein | 0.59 | 0.76 | -0.76 | -1.89 | N/A | N/A | No |
| 3 | PF3D7_0202400 | Gamete antigen 27/25, putative (translation enhancing factor plasmoDB) | 0.55 | 1.17 | -1.17 | -1.46 | ribosome binding | cytoplasm;host cell;host cell cytoplasm;plasma membrane;symbiont-containing vacuole | No |
| 4 | PF3D7_1368100 | 26S proteasome regulatory subunit RPN11,putative | 0.55 | 1.71 | -1.71 | -0.45 | metallopeptidase activity;proteasome binding;thiol-dependent deubiquitinase | nucleus;proteasome regulatory particle;proteasome regulatory particle, lid subcomplex | Yes |
| 5 | PF3D7_1208600 | Mitochondrial import inner membrane translocase subunit TIM10, putative | 0.55 | 2.26 | -2.26 | -0.92 | protein-transporting ATPase activity | TIM23 mitochondrial import inner membrane translocase complex;mitochondrial inner membrane;mitochondrial intermembrane space;mitochondrion | Likely yes |
| 6 | PF3D7_1312500 | Uncharacterized protein | 0.54 | 2.26 | -2.26 | n.d. | N/A | N/A | No |
| 7 | PF3D7_1140100 | V-type proton ATPase subunit F | 0.52 | 1.25 | -1.25 | -1.22 | ATPase-coupled ion transmembrane transporter activity;proton-transporting ATPase activity, rotational mechanism | membrane;mitochondrial proton-transporting ATP synthase complex;nucleus;vacuolar proton-transporting V-type ATPase, V1 domain | Unknown |
| 8 | PF3D7_0917900 | Heat shock protein 70 (BIP) | 0.52 | 1.11 | -1.11 | -1.24 | ATP binding;ATP hydrolysis activity;RNA binding;heat shock protein binding;misfolded protein binding;protein binding;protein folding chaperone;unfolded protein binding | cell surface;cytoplasm;endoplasmic reticulum;endoplasmic reticulum chaperone complex;endoplasmic reticulum lumen;extracellular vesicle;food vacuole;membrane;nucleus | Likely yes |
| 9 | PF3D7_1343700 | Kelch protein K13 | 0.51 | 1.28 | -1.28 | -0.77 | protein binding | cytoplasm;cytosol;endoplasmic reticulum;vesicle | Yes |
| 10 | PF3D7_0831700 | Heat shock protein 70 | 0.51 | 2.07 | -2.07 | -0.01 | ATP binding;ATP hydrolysis activity;RNA binding;heat shock protein binding;misfolded protein binding;protein binding;protein folding chaperone;unfolded protein binding | Maurer's cleft;cytoplasm;extracellular vesicle;host cell cytoplasm;host cell cytosol;nucleus;protein-containing complex;symbiont-containing vacuole | No |
| 11 | PF3D7_1371900 | Uncharacterized protein | 0.50 | 0.99 | -0.99 | -0.02 | N/A | host cell | Likely no |
| 12 | PF3D7_0822100 | Mediator of RNA polymerase II transcription subunit 7 | 0.50 | 1.87 | -1.87 | -0.11 | N/A | core mediator complex;mediator complex | Likely yes |

Characteristics of proteins identified as translationally increased with Lys codon bias in **Fig. 3c and Source Data Fig. 3**. Numbers correspond to those numbers on individual protein circles in **Fig. 3c**. Protein fold change values have been log2 transformed. LysAAA and LysAAG codon usage for each protein was determined using a codon counting algorithm (**Source Data Fig. 3**). Cutoff for LysAAA codon bias was Z scores >0.5 (proteins shown in red). Transcriptomic data were analyzed to determine fold change values of $Dd2^{R539T}$ parasites from time 0 versus a 6 hr DHA pulse. Values are log2 transformed. Proteins where the transcriptomic fold change was decreased despite increased protein amounts were considered. Top associated GO terms and GO localization were determined from PlasmoDB and noted next to the gene. Essentiality was also determined through PlasmoDB and shown graphically in **Extended Data Fig. 3d.**

**Supplementary Table 4.** Decreased proteins in Dd2$^{R539T}$ parasites post-DHA pulse regulated by Lys codon bias translation.

| Number | Gene ID | Gene Name | Log2 Protein Fold Change | LysAAA Z score | LysAAG Z score | Log2 RNA Fold Change | Top associated GO terms | GO Localization | Essential |
|---|---|---|---|---|---|---|---|---|---|
| 13 | PF3D7_1460600 | Inner membrane complex sub-compartment protein 3 | -0.50 | -1.46 | 1.46 | 0.40 | N/A | cell periphery;inner membrane pellicle complex | Unknown |
| 14 | PF3D7_1142500 | 60S ribosomal protein L28 | -0.51 | -0.99 | 0.99 | 0.47 | N/A | cytosolic large ribosomal subunit;nucleus | Likely yes |
| 15 | PF3D7_1310800 | Uncharacterized protein | -0.51 | -0.62 | 0.62 | 0.10 | N/A | N/A | Yes |
| 16 | PF3D7_1357000 | Elongation factor 1-alpha | -0.52 | -1.54 | 1.54 | n.d. | GTPase activity;translation elongation factor activity | eukaryotic translation elongation factor 1 complex;infected host cell surface knob;mitochondrion;nucleus | Likely yes |
| 17 | PF3D7_1453700 | Co-chaperone p23 | -0.53 | -1.77 | 1.77 | 2.09 | Hsp90 protein binding;chaperone binding | cytoplasm;cytosol;nucleus | Unclear |
| 18 | PF3D7_1408600 | 40S ribosomal protein S8 | -0.55 | -1.54 | 1.54 | 2.77 | RNA binding;structural constituent of ribosome | cytosolic small ribosomal subunit;nucleus | No |
| 19 | PF3D7_0415900 | Ribosomal protein L15 | -0.61 | -0.76 | 0.76 | 0.42 | RNA binding;structural constituent of ribosome | cytosolic large ribosomal subunit;nucleus;ribosome | Yes |
| 20 | PF3D7_0802000 | Glutamate dehydrogenase, putative | -0.62 | -0.60 | 0.60 | 0.10 | glutamate dehydrogenase (NAD+) activity | cytosol;mitochondrion;nucleus | Likely no |
| 21 | PF3D7_1317800 | 40S ribosomal protein S19 | -0.64 | -1.77 | 1.77 | 0.33 | RNA binding;structural constituent of ribosome | cytosolic small ribosomal subunit;nucleus | Yes |
| 22 | PF3D7_1321200 | Uncharacterized protein | -0.65 | -0.76 | 0.76 | 1.95 | N/A | N/A | Yes |

Characteristics of proteins identified as translationally decreased with Lys codon bias in **Fig. 3c and Source Data Fig. 3**. Numbers correspond to those numbers on individual protein circles in **Fig. 3c**. Protein fold change values have been log2 transformed. LysAAA and LysAAG codon usage for each protein was determined using a codon counting algorithm (**Source Data Fig. 3**). Cutoff for LysAAA codon bias was Z scores >0.5 (proteins shown in red). Transcriptomic data were analyzed to determine fold change values of Dd2$^{R539T}$ parasites from time 0 versus a 6 hr DHA pulse. Values are log2 transformed. Proteins where the transcriptomic fold change was decreased despite increased protein amounts were considered. Top associated GO terms and GO localization were determined from PlasmoDB and noted next to the gene. Essentiality was also determined through PlasmoDB and shown graphically in **Extended Data Fig. 3d.**

**Supplementary Table 5.** Increased proteins in $Dd2^{R539T}$ parasites post-DHA pulse regulated by His codon bias translation.

| Gene ID | Gene Name | Log2 Protein Fold Change | HisCAT Z score | HisCAC Z score | Log2 RNA Fold Change | Top associated GO terms | GO Localization | Essential |
|---|---|---|---|---|---|---|---|---|
| PF3D7_1208600 | Mitochondrial import inner membrane translocase subunit TIM10, putative | 0.55 | 0.94 | -0.94 | -0.92 | protein-transporting ATPase activity | TIM23 mitochondrial import inner membrane translocase complex;mitochondrial inner membrane;mitochondrial intermembrane space;mitochondrion | Likely yes |
| PF3D7_1312500 | Uncharacterized protein | 0.54 | 0.94 | -0.94 | n.d. | N/A | N/A | No |
| PF3D7_0933900 | Uncharacterized protein | 0.54 | 0.94 | -0.94 | -5.10 | N/A | nucleus | No |
| PF3D7_1001000 | Uncharacterized protein | 0.54 | 0.94 | -0.94 | n.d. | protein binding | Maurer's cleft;host cell surface;infected host cell surface knob | Unclear |
| PF3D7_1140100 | V-type proton ATPase subunit F | 0.52 | 0.94 | -0.94 | -1.22 | ATPase-coupled ion transmembrane transporter activity;proton-transporting ATPase activity, rotational mechanism | membrane;mitochondrial proton-transporting ATP synthase complex;nucleus;vacuolar proton-transporting V-type ATPase, V1 domain | Unclear |
| PF3D7_1371900 | Uncharacterized protein | 0.50 | 0.94 | -0.94 | -0.02 | N/A | host cell | Likely no |

Characteristics of proteins identified as translationally increased with His codon bias in **Extended Data Fig 3b and Source Data Extended Data Fig. 3**. Protein fold change values have been log2 transformed. HisCAT and HisCAC codon usage for each protein was determined using a codon counting algorithm (**Source Data Fig. 3**). Cutoff for HisCAT codon bias was Z scores >0.5 (proteins shown in red). Transcriptomic data were analyzed to determine fold change values of $Dd2^{R539T}$ parasites from time 0 versus a 6 hr DHA pulse. Values are log2 transformed. Proteins where the transcriptomic fold change was decreased despite increased protein amounts were considered. Top associated GO terms and GO localization were determined from PlasmoDB and noted next to the gene. Essentiality was also determined through PlasmoDB.

**Supplementary Table 6.** Decreased proteins in Dd2$^{R539T}$ parasites post-DHA pulse regulated by His codon bias translation.

| Gene ID | Gene Name | Log2 Protein Fold Change | HisCAT Z score | HisCAC Z score | Log2 RNA Fold Change | Top associated GO terms | GO Localization | Essential |
|---|---|---|---|---|---|---|---|---|
| PF3D7_1460600 | Inner membrane complex sub-compartment protein 3 | -0.50 | -4.93 | 4.93 | 0.40 | N/A | cell periphery;inner membrane pellicle complex | Unclear |
| PF3D7_1142500 | 60S ribosomal protein L28 | -0.51 | -1.02 | 1.02 | 0.47 | N/A | cytosolic large ribosomal subunit;nucleus | Likely yes |
| PF3D7_1357000 | Elongation factor 1-alpha | -0.52 | -4.27 | 4.27 | n.d. | GTPase activity;translation elongation factor activity | eukaryotic translation elongation factor 1 complex;infected host cell surface knob;mitochondrion;nucleus | Likely yes |
| PF3D7_1453700 | Co-chaperone p23 | -0.53 | -4.93 | 4.93 | 2.09 | Hsp90 protein binding;chaperone binding | cytoplasm;cytosol;nucleus | Unclear |
| PF3D7_1408600 | 40S ribosomal protein S8 | -0.55 | -4.93 | 4.93 | 2.77 | RNA binding;structural constituent of ribosome | cytosolic small ribosomal subunit;nucleus | No |
| PF3D7_0306200 | Activator of Hsp90 ATPase | -0.57 | -1.99 | 1.99 | n.d. | ATPase activator activity;Hsp90 protein binding | cytosol | Likely no |
| PF3D7_0415900 | Ribosomal protein L15 | -0.61 | -1.02 | 1.02 | 0.42 | RNA binding;structural constituent of ribosome | cytosolic large ribosomal subunit;nucleus;ribosome | Yes |
| PF3D7_0813000 | Uncharacterized protein | -0.70 | -0.74 | 0.74 | 1.50 | N/A | cytoplasm;cytosol | Likely yes |

Characteristics of proteins identified as translationally increased with His codon bias in **Extended Data Fig. 3b and Source Data Extended Data Fig. 3**. Protein fold change values have been log2 transformed. HisCAT and HisCAC codon usage for each protein was determined using a codon counting algorithm (**Source Data Fig. 3**). Cutoff for HisCAC codon bias was Z scores >0.5 (proteins shown in red). Transcriptomic data were analyzed to determine fold change values of Dd2$^{R539T}$ parasites from time 0 versus a 6 hr DHA pulse. Values are log2 transformed. Proteins where the transcriptomic fold change was decreased despite increased protein amounts were considered. Top associated GO terms and GO localization were determined from PlasmoDB and noted next to the gene. Essentiality was also determined through PlasmoDB**.**

**Supplementary Table 7.** Increased proteins in Dd2$^{R539T}$ parasites post-DHA pulse regulated by Asp codon bias translation.

| Gene ID | Gene Name | Log2 Protein Fold Change | AspGAT Z score | AspGAC Z score | Log2 RNA Fold Change | Top associated GO terms | GO Localization | Essential |
|---|---|---|---|---|---|---|---|---|
| PF3D7_0625400 | Uncharacterized protein | 0.63 | 0.79 | -0.79 | -0.26 | N/A | N/A | Yes |
| PF3D7_1368100 | 26S proteasome regulatory subunit RPN11,putative | 0.55 | 0.67 | -0.67 | -0.45 | metallopeptidase activity;proteasome binding;thiol-dependent deubiquitinase | nucleus;proteasome regulatory particle;proteasome regulatory particle, lid subcomplex | Yes |
| PF3D7_1329300 | Chromatin assembly factor 1 subunit, putative | 0.55 | 0.53 | -0.53 | -2.65 | chromatin binding;histone binding;protein binding;unfolded protein binding | nucleus | Likely yes |
| PF3D7_1001000 | Uncharacterized protein | 0.54 | 0.77 | -0.77 | n.d. | protein binding | Maurer's cleft;host cell surface;infected host cell surface knob | Unclear |
| PF3D7_0424600 | Uncharacterized protein | 0.54 | 1.36 | -1.36 | -0.26 | N/A | extracellular vesicle;host cell periphery;host cell surface;nucleus | Likely no |
| PF3D7_1343700 | Kelch protein K13 | 0.51 | 1.36 | -1.36 | -0.77 | protein binding | cytoplasm;cytosol;endoplasmic reticulum;vesicle | Yes |
| PF3D7_0831700 | Heat shock protein 70 | 0.51 | 1.17 | -1.17 | -0.01 | ATP binding;ATP hydrolysis activity;RNA binding;heat shock protein binding;misfolded protein binding;protein binding;protein folding chaperone;unfolded protein binding | Maurer's cleft;cytoplasm;extracellular vesicle;host cell cytoplasm;host cell cytosol;nucleus;protein-containing complex;symbiont-containing vacuole | No |
| PF3D7_0822100 | Mediator of RNA polymerase II transcription subunit 7 | 0.50 | 0.80 | -0.80 | -0.11 | N/A | core mediator complex;mediator complex | Likely yes |

Characteristics of proteins identified as translationally increased with Asp codon bias in **Extended Data Fig. 3c and Source Data Extended Data Fig. 3**. Protein fold change values have been log2 transformed. AspGAT and AspGAC codon usage for each protein was determined using a codon counting algorithm (**Source Data Fig. 3**). Cutoff for AspGAT codon bias was Z scores >0.5 (proteins shown in red). Transcriptomic data were analyzed to determine fold change values of Dd2$^{R539T}$ parasites from time 0 versus a 6 hr DHA pulse. Values are log2 transformed. Proteins where the transcriptomic fold change was decreased despite increased protein amounts were considered. Top associated GO terms and GO localization were determined from PlasmoDB and noted next to the gene. Essentiality was also determined through PlasmoDB**.**

**Supplementary Table 8.** Decreased proteins in Dd2$^{R539T}$ parasites post-DHA pulse regulated by Asp codon bias translation.

| Gene ID | Gene Name | Log2 Protein Fold Change | AspGAT Z score | AspGAC Z score | Log2 RNA Fold Change | Top associated GO terms | GO Localization | Essential |
|---|---|---|---|---|---|---|---|---|
| PF3D7_1460600 | Inner membrane complex sub-compartn | -0.50 | -0.63 | 0.63 | 0.40 | N/A | cell periphery;inner membrane pellicle complex | Unclear |
| PF3D7_0813900 | 40S ribosomal protein S16, putative | -0.51 | -1.62 | 1.62 | n.d | RNA binding;structural constituent of ribosome | cytosolic small ribosomal subunit;nucleus;small ribosomal subunit | Yes |
| PF3D7_1142500 | 60S ribosomal protein L28 | -0.51 | -3.11 | 3.11 | 0.47 | N/A | cytosolic large ribosomal subunit;nucleus | Likely yes |
| PF3D7_1357000 | Elongation factor 1-alpha | -0.52 | -0.58 | 0.58 | n.d. | GTPase activity;translation elongation factor activity | eukaryotic translation elongation factor 1 complex;infected host cell surface knob;mitochondrion;nucleus | Likely yes |
| PF3D7_1237400 | Uncharacterized protein | -0.80 | -1.62 | 1.62 | 0.86 | N/A | N/A | No |

Characteristics of proteins identified as translationally increased with Asp codon bias in **Extended Data Fig. 3c and Source Data Extended Data Fig. 3**. Protein fold change values have been log2 transformed. AspGAT and AspGAC codon usage for each protein was determined using a codon counting algorithm (**Source Data Fig. 3**). Cutoff for AspGAC codon bias was Z scores >0.5 (proteins shown in red). Transcriptomic data were analyzed to determine fold change values of Dd2$^{R539T}$ parasites from time 0 versus a 6 hr DHA pulse. Values are log2 transformed. We considered proteins where the transcriptomic fold change was decreased despite increased protein amounts. Top associated GO terms and GO localization were determined from PlasmoDB and noted next to the gene. Essentiality was also determined through PlasmoDB**.**

**Supplementary Table 9.** Codon use bias between up and down regulated proteins in Dd2$^{R539T}$ parasites post DHA pulse likely reguated by Lys, His and/or Asp codon bias translation.

| Differentially Increased Proteins with Codon Bias | | | | | | Differentially Decreased Proteins with Codon Bias | | | | | |
|---|---|---|---|---|---|---|---|---|---|---|---|
| **Gene ID** | **Gene Name** | **LysAAA** | **HisCAT** | **AspGAT** | **Essential** | **Gene ID** | **Gene Name** | **LysAAG** | **HisCAC** | **AspGAC** | **Essential** |
| PF3D7_0625400 | Uncharacterized protein | **x** | | **x** | yes | PF3D7_1460600 | Inner membrane complex sub-compartment protein 3 | **x** | **x** | **x** | unknown |
| PF3D7_1368100 | 26S proteasome regulatory subunit RPN11,putative | **x** | | **x** | yes | PF3D7_1142500 | 60S ribosomal protein L28 | **x** | **x** | **x** | likely yes |
| PF3D7_1208600 | Mitochondrial import inner membrane translocase subunit TIM10, putative | **x** | **x** | | likely yes | PF3D7_1357000 | Elongation factor 1-alpha | **x** | **x** | **x** | likely yes |
| PF3D7_1312500 | Uncharacterized protein | **x** | **x** | | no | PF3D7_1453700 | Co-chaperone p23 | **x** | **x** | | unknown |
| PF3D7_1140100 | V-type proton ATPase subunit F | **x** | **x** | | unknown | PF3D7_1408600 | 40S ribosomal protein S8 | **x** | **x** | | no |
| PF3D7_1343700 | Kelch protein K13 | **x** | | **x** | yes | PF3D7_0415900 | Ribosomal protein L15 | **x** | **x** | | yes |
| PF3D7_0831700 | Heat shock protein 70 | **x** | | **x** | no | | | | | | |
| PF3D7_1371900 | Uncharacterized protein | **x** | **x** | | likely no | | | | | | |
| PF3D7_0822100 | Mediator of RNA polymerase II transcription subunit 7 | **x** | | **x** | likely yes | | | | | | |
| PF3D7_1001000 | Uncharacterized protein | | **x** | **x** | unknown | | | | | | |

Characteristics of proteins identified as regulated by codon bias translation in **Supplementary Tables 3-8**. Xs indicate which codons had usage bias in each protein. Essentiality was also determined through PlasmoDB**.**

**Supplementary Table 10.** List of oligonucleotides used in this study.

| Name | Nucleotide sequence | Description | Lab name |
|---|---|---|---|
| p1 | ggtgtacggtacaaacccggaattcgagctcggCCAGATAAATGA TTTAAACATGAGC | Pf1019800 RHR fw into pSN054 | p8077 |
| p2 | tggataagacgagagattgggtattagacctagggataacagggtaatga atttatgcggataaatgatatgaa | Pf1019800 RHR rev into pSN054 | p8078 |
| p3 | attggttttcaaacttcattgactgtgccggccggccGAAACCAAAGG TCTCTGT | Pf1019800 LHR fw pSN054 to combine with recoded LHR and into pSN054 vector | p8079 |
| p4 | TTGCTTTTTTATACATGCAGGTAATTTCCCCTCTAT CCATTTTATATTGTGCAATTTAC | Pf1019800 LHR rev pSN054 to combine with recoded LHR and into pSN054 vector | p8080 |
| p5 | taatacgactcactataggTATAAGGTGATAATTTGTCCgtttt agagctagaaatag | pf1019800 guide 1 into pSN054 fw | p8105 |
| p6 | ctatttctagctctaaaacGGACAAATTATCACCTTATAcctata gtgagtcgtatta | pf1019800 guide 1 into pSN054 rev | p8106 |
| p7 | ataatacgactcactataggAAAGATACCGGCTTTTCACCgtt ttagagctagaaata | pf1019800 guide 2 into pSN054 fw | p8107 |
| p8 | tatttctagctctaaaacGGTGAAAAGCCGGTATCTTTcctat agtgagtcgtattat | pf1019800 guide 2 into pSN054 rev | p8108 |
| p9 | GGTCTCGTTCGTCTTGTACTTG | pSN054 seq primer pf1019800 specific | p8111 |
| p10 | cggcctaactgtggccag | pSN054 seq primer LHR fw universal | p8112 |
| p11 | CGcTGtcacagatcttcttcg | pSN054 seq primer LHR rev universal | p8113 |
| p12 | cagtggtgtacggtacaaacccg | pSN054 seq primer RHR rev universal | p8114 |
| p13 | cgggtttgtaccgtacaccactg | gRNA seq for pSN054 vector | p8188 |
| p14 | tgtagtagatgtgattgaatcatg | Pf1019800 cKD internal genome integration check rev | p8244 |
| p15 | gccctcccacacataaccagagggcag | Pf1019800 cKD internal genome integration check fw | p8245 |
| p16 | GATCAGCAGCTTCTATAACATAATCCC | Pf1019800 RHR genome integration check rev | p8246 |
| p17 | CTGGACATTATGCTATGATAAGAACGAATG | Pf1019800 LHR genome integration check fw | p8249 |
| p18 | GGTCTTCTCGTCGTGTGGTATG | Pf1019800 LHR genome integration check rev | p8259 |

Raw data for morphology analysis of PfMnmA conditional knockdown cultured ± aTc for 168 hr then recultured ± aTc.

| | 72 hr | | | | | 96 hr | | | | | 120 hr | | | | | 144 hr | | | | | 168 hr | | | | | 240 hr | | | | |
|---|---|---|---|---|---|---|---|---|---|---|---|---|---|---|---|---|---|---|---|---|---|---|---|---|---|---|---|---|---|---|
| **Line** | Ring | Troph | Schizont | RBC | Total | Ring | Troph | Schizont | RBC | Total | Ring | Troph | Schizont | RBC | Total | Ring | Troph | Schizont | RBC | Total | Ring | Troph | Schizont | RBC | Total | Ring | Troph | Schizont | RBC | Total |
| PfMnmA_cKD +aTc, +aTc | 2.98 | 0.00 | 0.89 | 100.00 | 3.87 | 1.11 | 2.22 | 1.11 | 100.00 | 4.44 | 7.73 | 1.60 | 0.53 | 100.00 | 9.87 | 2.08 | 0.26 | 0.78 | 100.00 | 3.12 | 3.91 | 1.68 | 0.28 | 100.00 | 5.87 | 3.55 | 0.71 | 0.71 | 100.00 | 4.96 |
| PfMnmA_cKD +aTc, -aTc | 2.46 | 0.82 | 0.27 | 100.00 | 3.55 | 1.43 | 1.43 | 0.86 | 100.00 | 3.72 | 5.52 | 0.29 | 0.00 | 100.00 | 5.81 | 0.73 | 0.73 | 0.24 | 100.00 | 1.71 | 1.01 | 0.25 | 0.00 | 100.00 | 1.26 | 0.92 | 0.00 | 0.00 | 100.00 | 0.92 |
| PfMnmA_cKD -aTc, +aTc | 0.84 | 0.00 | 0.00 | 100.00 | 0.84 | 0.55 | 0.83 | 0.00 | 100.00 | 1.39 | 1.52 | 0.00 | 0.00 | 100.00 | 1.52 | 0.48 | 0.24 | 0.48 | 100.00 | 1.21 | 1.55 | 0.52 | 0.26 | 100.00 | 2.32 | 3.29 | 0.55 | 0.27 | 100.00 | 4.11 |
| PfMnmA_cKD -aTc, -aTc | 0.00 | 0.00 | 0.00 | 100.00 | 0.00 | 0.00 | 0.00 | 0.00 | 100.00 | 0.00 | 0.33 | 0.00 | 0.00 | 100.00 | 0.33 | 0.26 | 0.00 | 0.00 | 100.00 | 0.26 | 0.61 | 0.00 | 0.00 | 100.00 | 0.61 | 0.31 | 0.00 | 0.00 | 100.00 | 0.31 |

Raw data for morphology studies demonstrated in **Supplementary Fig. 1.** NF54_Crispr_T7 pol parasites and PfMnmA_cKD parasites from 168 hr shown in **Fig. 4c** and **Source Data Fig. 4** were then thoroughly washed and split again into media with or without 500 nM aTc. Thin smears were made and Geimsa stained at the indicated time points. 100 RBC were counted per slide. Numbers represent the percent of parasites that were rings, trophozoites (troph), schizonts and the total parasitemia. n=1 biologic replicate

Ring stage survival assay values of PfMnmA_cKD parasite lines cultured with aTc prior to chloroquine drug pulse.

| | NF54, 30 nM aTc | PfMnmA_cKD, 30 nM aTc | NF54, 0 nM aTc | PfMnmA_cKD, 0 nM aTc |
|---|---|---|---|---|
| **300 nM** | **37 ± 8.9** | **44 ± 6.6** | **35 ± 7.5** | **40 ± 6.1** |
| N | 4 | 4 | 4 | 4 |
| *P* vs NF54 at same aTc | – | 0.4857 | – | 0.6857 |
| *P* vs 30 nM aTc | – | – | 0.8857 | 0.4857 |
| **150 nM** | **49 ± 2.8** | **53 ± 5.5** | **50 ± 2.8** | **54 ± 3.8** |
| N | 4 | 4 | 4 | 4 |
| *P* vs NF54 at same aTc | – | 0.6857 | – | 0.6857 |
| *P* vs 30 nM aTc | – | – | 0.8857 | >0.9999 |
| **75 nM** | **54 ± 4.5** | **62 ± 2.8** | **56 ± 4.6** | **60 ± 2.9** |
| N | 4 | 4 | 4 | 4 |
| *P* vs NF54 at same aTc | – | 0.4857 | – | 0.6857 |
| *P* vs 30 nM aTc | – | – | 0.6857 | >0.9999 |
| **37.5 nM** | **56 ± 4.4** | **63 ± 3.9** | **63 ± 4.5** | **63 ± 2.0** |
| N | 4 | 4 | 4 | 4 |
| *P* vs NF54 at same aTc | – | 0.3429 | – | 0.8857 |
| *P* vs 30 nM aTc | – | – | 0.4857 | 0.8857 |
| **18.75 nM** | **63 ± 4.3** | **71 ± 3.5** | **70 ± 5.4** | **72 ± 1.2** |
| N | 4 | 4 | 4 | 4 |
| *P* vs NF54 at same aTc | – | 0.2 | – | >0.9999 |
| *P* vs 30 nM aTc | – | – | 0.4857 | 0.8857 |
| **9.375 nM** | **75 ± 4.7** | **83 ± 2.6** | **84 ± 4.0** | **78 ± 3.1** |
| N | 4 | 4 | 4 | 4 |
| *P* vs NF54 at same aTc | – | 0.2 | – | 0.4857 |
| *P* vs 30 nM aTc | – | – | 0.2 | 0.2 |
| **4.69 nM** | **90 ± 3.0** | **93 ± 2.8** | **92 ± 1.5** | **90 ± 2.6** |
| N | 4 | 4 | 4 | 4 |
| *P* vs NF54 at same aTc | – | 0.6857 | – | 0.6857 |
| *P* vs 30 nM aTc | – | – | 0.6857 | 0.3429 |
| **2.34 nM** | **93 ± 1.4** | **93 ± 1.9** | **95 ± 2.3** | **94 ± 1.6** |
| N | 4 | 4 | 4 | 4 |
| *P* vs NF54 at same aTc | – | 0.8857 | – | 0.4857 |
| *P* vs 30 nM aTc | – | – | 0.3429 | >0.9999 |
| **1.17 nM** | **93 ± 1.7** | **96 ± 1.6** | **96 ± 1.5** | **92 ± 1.7** |
| N | 4 | 4 | 4 | 4 |
| *P* vs NF54 at same aTc | – | 0.4857 | – | 0.2 |
| *P* vs 30 nM aTc | – | – | 0.4857 | 0.2 |
| **0.59 nM** | **98 ± 1.1** | **96 ± 1.0** | **97 ± 0.27** | **95 ± 1.3** |
| N | 4 | 4 | 4 | 4 |
| *P* vs NF54 at same aTc | – | 0.2 | – | 0.0286 |
| *P* vs 30 nM aTc | – | – | 0.4857 | 0.6857 |

Ring-stage survival assay (RSA) values (nM) indicate the mean ± SEM, as determined in 4 independent assays performed in duplicate (**Supplementary Figure 2b**). Parasite survival is defined as the ratio of the parasitemias of the chloroquine (CQ)-treated to the no-drug control wells. This assay measures the survival of synchronous ring-stage parasites (0-6 hr post-invasion) exposed to CQ for 6 hr, washed three times and then allowed to recover for 72 hr prior to measuring parasitemias by flow cytometry. N, number of independent assays. Statistical significance was determined via two-tailed Mann-Whitney U tests. *P* values are reported for comparisons with the parasite lines NF54 or the PfMnmA_cKD outgrown with 30 nM aTc.  All parasites in this assay were cultured with aTc and washed thoroughly prior to the start of the assay.

Ring-stage survival assay values of PfMnmA_cKD parasite lines cultured without aTc prior to chloroquine drug pulse.

| | NF54, 30 nM aTc | PfMnmA_cKD, 30 nM aTc | NF54, 0 nM aTc | PfMnmA_cKD, 0 nM aTc |
|---|---|---|---|---|
| **300 nM** | **37 ± 8.9** | **44 ± 6.6** | **35 ± 7.5** | **40 ± 6.1** |
| N | 4 | 4 | 4 | 4 |
| *P* vs NF54 at same aTc | – | 0.4857 | – | 0.6857 |
| *P* vs 30 nM aTc | – | – | 0.8857 | 0.4857 |
| **150 nM** | **47 ± 0.76** | **52 ± 4.1** | **47 ± 3.0** | **47 ± 8.0** |
| N | 4 | 4 | 4 | 4 |
| *P* vs NF54 at same aTc | – | 0.3429 | – | 0.8857 |
| *P* vs 30 nM aTc | – | – | 0.3429 | 0.6857 |
| **75 nM** | **54 ± 3.5** | **53 ± 6.0** | **54 ± 3.4** | **52 ± 7.6** |
| N | 4 | 4 | 4 | 4 |
| *P* vs NF54 at same aTc | – | 0.8857 | – | 0.6857 |
| *P* vs 30 nM aTc | – | – | 0.8857 | 0.8857 |
| **37.5 nM** | **58 ± 2.3** | **58 ± 5.4** | **59 ± 2.5** | **54 ± 7.0** |
| N | 4 | 4 | 4 | 4 |
| *P* vs NF54 at same aTc | – | >0.9999 | – | 0.8857 |
| *P* vs 30 nM aTc | – | – | 0.4857 | 0.6857 |
| **18.75 nM** | **66 ± 2.3** | **69 ± 4.4** | **72 ± 3.3** | **65 ± 4.0** |
| N | 4 | 4 | 4 | 4 |
| *P* vs NF54 at same aTc | – | 0.6857 | – | 0.3429 |
| *P* vs 30 nM aTc | – | – | 0.2 | 0.6857 |
| **9.375 nM** | **80 ± 1.8** | **76 ± 5.9** | **82 ± 4.0** | **83 ± 4.8** |
| N | 4 | 4 | 4 | 4 |
| *P* vs NF54 at same aTc | – | 0.8857 | – | >0.9999 |
| *P* vs 30 nM aTc | – | – | 0.6857 | 0.6857 |
| **4.69 nM** | **88 ± 1.2** | **84 ± 1.7** | **85 ± 0.87** | **86 ± 3.0** |
| N | 4 | 4 | 4 | 4 |
| *P* vs NF54 at same aTc | – | 0.1143 | – | 0.8857 |
| *P* vs 30 nM aTc | – | – | 0.2 | 0.6857 |
| **2.34 nM** | **92 ± 1.2** | **89 ± 2.5** | **93 ± 1.2** | **90 ± 4.1** |
| N | 4 | 4 | 4 | 4 |
| *P* vs NF54 at same aTc | – | 0.3429 | – | >0.9999 |
| *P* vs 30 nM aTc | – | – | 0.8857 | 0.8857 |
| **1.17 nM** | **91 ± 3.2** | **97 ± 1.4** | **96 ± 0.78** | **84 ± 2.7** |
| N | 4 | 4 | 4 | 4 |
| *P* vs NF54 at same aTc | – | 0.2 | – | 0.0286 |
| *P* vs 30 nM aTc | – | – | 0.3429 | 0.0286 |
| **0.59 nM** | **94 ± 2.0** | **87 ± 4.3** | **95 ± 1.7** | **93 ± 1.4** |
| N | 4 | 4 | 4 | 4 |
| *P* vs NF54 at same aTc | – | 0.3429 | – | 0.6857 |
| *P* vs 30 nM aTc | – | – | 0.6857 | 0.2 |

Ring-stage survival assay (RSA) values (nM) indicate the mean ± SEM, as determined in 4 independent assays performed in duplicate (**Supplementary Fig. 2a**). Parasite survival is defined as the ratio of the parasitemias of the chloroquine (CQ)-treated to the no-drug control wells. This assay measures the survival of synchronous ring-stage parasites (0-6 hr post-invasion) exposed to CQ for 6 hr, washed three times and then allowed to recover for 72 hr prior to measuring parasitemias by flow cytometry. N, number of independent assays. Statistical significance was determined via two-tailed Mann-Whitney U tests. *P* values are reported for comparisons with the parasite lines NF54 or the PfMnmA_cKD outgrown with 30 nM aTc. All parasites in this assay were grown with aTc and washed thoroughly prior to the start of the assay.
