# Supplementary material for: tRNA modification reprogramming contributes to artemisinin resistance in Plasmodium falciparum
Source: Nat Microbiol. 2024 Apr 17;9(6):1483–98. doi: 10.1038/s41564-024-01664-3 (PMC11153160; doi:10.1038/s41564-024-01664-3)
Supplement: Supplementary file 2 — Reporting Summary [file 41564_2024_1664_MOESM2_ESM.pdf]

nature portfolio

Corresponding author(s): David A. Fidock, PhD
Jennifer Small-Saunders, MD, PhD

Last updated by author(s): Feb 15, 2024

Reporting Summary

Nature Portfolio wishes to improve the reproducibility of the work that we publish. This form provides structure for consistency and transparency in reporting. For further information on Nature Portfolio policies, see our Editorial Policies and the Editorial Policy Checklist.

Statistics

For all statistical analyses, confirm that the following items are present in the figure legend, table legend, main text, or Methods section.

n/a	Confirmed	
☐	☒	The exact sample size (*n*) for each experimental group/condition, given as a discrete number and unit of measurement
☐	☒	A statement on whether measurements were taken from distinct samples or whether the same sample was measured repeatedly
☐	☒	The statistical test(s) used AND whether they are one- or two-sided<br>*Only common tests should be described solely by name; describe more complex techniques in the Methods section.*
☐	☒	A description of all covariates tested
☐	☒	A description of any assumptions or corrections, such as tests of normality and adjustment for multiple comparisons
☐	☒	A full description of the statistical parameters including central tendency (e.g. means) or other basic estimates (e.g. regression coefficient) AND variation (e.g. standard deviation) or associated estimates of uncertainty (e.g. confidence intervals)
☐	☒	For null hypothesis testing, the test statistic (e.g. *F*, *t*, *r*) with confidence intervals, effect sizes, degrees of freedom and *P* value noted<br>*Give P values as exact values whenever suitable.*
☒	☐	For Bayesian analysis, information on the choice of priors and Markov chain Monte Carlo settings
☒	☐	For hierarchical and complex designs, identification of the appropriate level for tests and full reporting of outcomes
☒	☐	Estimates of effect sizes (e.g. Cohen's *d*, Pearson's *r*), indicating how they were calculated

Our web collection on statistics for biologists contains articles on many of the points above.

Software and code

Policy information about availability of computer code

Data collection	Proteomics: QExactive HF-X mass spectrometer (Thermo Scientific), software: Thermo Xcalibur (Thermo)<br>tRNA modifications analysis: Agilent 6490 triple quadrupole mass spectrometer (LC-MS/MS) with ESI jetstreatm ioniziation, software: Mass Hunter Work Station Data Acquisition(Agilent)<br>Drug inhibition assays: FlowJo Version 10 (FlowJo LLC)
Data analysis	Proteomics: Raw mass spectral data files (.raw) searched using Proteome Discoverer (Thermo Scientific) and Mascot version 2.4.1 (Matrix Science). Quantification and stat testing of TMT proteomics: MSstats in R<br>tRNA modifications: Mass Hunter Quantitative Analysis and Qualitative Analysis (B.06) software (Agilent) and Microsoft Excel.<br>Principal component regression: Prism version 9 (GraphPad)<br>Lysine codon usage in PfK13 gene: Lasergene 17 (DNASTAR)<br>Drug inhibition assays: Prism version 9 (GraphPad)<br>Codon optimized PfMnmA sequence for molecular cloning: www.genedesign.org/CodonJuggling

For manuscripts utilizing custom algorithms or software that are central to the research but not yet described in published literature, software must be made available to editors and reviewers. We strongly encourage code deposition in a community repository (e.g. GitHub). See the Nature Portfolio guidelines for submitting code & software for further information.

Data

Policy information about availability of data

All manuscripts must include a data availability statement. This statement should provide the following information, where applicable:

- Accession codes, unique identifiers, or web links for publicly available datasets
- A description of any restrictions on data availability
- For clinical datasets or third party data, please ensure that the statement adheres to our policy

All data sets generated in this study are available in the following:
TMT-tagged Proteomics data: PRIDE repository accession #PXD043747 (DOI:10.6019/PDX043747, Username: reviewer_pxd043747@ebi.ac.uk, Password:jZKeFyVs.)
Previously published transcriptomics data set: NCBI's Gene Expression Omnibus with the identifier GSE151189
P. falciparum Gene Ontology and gene essentiality predication: PlasmoDB Release 63
All other data supporting the findings of this study are available within the paper and its supplementary information.

Research involving human participants, their data, or biological material

Policy information about studies with human participants or human data. See also policy information about sex, gender (identity/presentation), and sexual orientation and race, ethnicity and racism.

Reporting on sex and gender	Human red blood cells were purchased from a commercial blood bank and are pooled from anonymous blood donors. There are no identifiers that would enable the donors to be identified. Therefore no data on sex and gender were collected.
Reporting on race, ethnicity, or other socially relevant groupings	Human red blood cells were purchased from a commercial blood bank and are pooled from anonymous blood donors. There are no identifiers that would enable the donors to be identified. Therefore no data on race, ethnicity or other socially relevant groups were collected.
Population characteristics	No data on population characteristics were collected.
Recruitment	Human red blood cells were purchased from a commercial blood bank and were pooled from anonymous blood donors. There are no identifiers that would enable the donors to be identified. Therefore there was no participant recruitment.
Ethics oversight	A research protocol (IRB-AAAC4249) was submitted by Dr. Fidock to the Institutional Review Board (IRB) at the Columbia University Irving Medical Center (CUIMC). This protocol was approved on 22 September 2022 by the IRB as "not human subjects research in accordance with the Code of Federal Regulations Title 45 – Public Welfare Department of Health and Human Services, Part 46 – Protection of Human Subjects".

Note that full information on the approval of the study protocol must also be provided in the manuscript.

Field-specific reporting

Please select the one below that is the best fit for your research. If you are not sure, read the appropriate sections before making your selection.

☒ Life sciences ☐ Behavioural & social sciences ☐ Ecological, evolutionary & environmental sciences

For a reference copy of the document with all sections, see nature.com/documents/nr-reporting-summary-flat.pdf

Life sciences study design

All studies must disclose on these points even when the disclosure is negative.

Sample size	For tRNA modification LC-MS/MS analysis, seven independent biological replicates were performed for each condition and time point. Given the nature of tRNA modifications and the technical limitations of generating large amount of early ring stage parasites, this number was chosen to ensure changes that were seen were consistent and reproducible. For proteomics LC-MS/MS experiments, three independent biological samples were collected for each condition and each time point to ensure data reproducibility. This enabled identification of consistent and reproducible proteomic changes. Three to seven independent biological replicates, each with two technical replicates, were performed for ring stage survival assays, drug inhibition assays and heat shock assays to allow for statistical analysis of the data. For LC-MS/MS identification of specific tRNA modifications in the parasite knockdown lines, two independent biological samples were collected for each condition and time point, with two technical replicates for each, to ensure reproducibility.
Data exclusions	All data sets were included for tRNA modification and proteomic analyses, with further data processing as noted in the Methods. For assays with conditional knockdown parasites, assays were excluded if there was no parasite expansion. Following initial quality control no other assays were excluded. For LC-MS/MS identification of specific tRNA modifications in the parasite knockdown lines, the 96 hr time point for replicate 1 was excluded as the RNA became degraded. This is noted in the text and figure legend.
Replication	For sample collection for tRNA modification (seven independent replicates) and proteomics (three independent replicates) analyses, Dd2 and Dd2_R539T parasites were always highly-synchonized to the same early ring stage and processed together for each biological replicate to minimize effects of any potential minor alterations in media or temperature and to ensure reproducibility. Samples for proteomic LC-MS/MS were multiplexed for each independent biological replicate to ensure reproducibility in sample to sample comparisons. All attempts were

	successful and no data was excluded. For ring stage survival assays, drug inhibition assays and growth assays with the conditional knockdown line, three to seven independent biological replicates were performed per sample, with two technical replicates, and every assay contained the parental NF54 control parasite along with the translation on and translation off knockdown. All attempts at replication were successful.
Randomization	Allocation of samples randomly into experimental groups was not relevant to this study as we were assaying isogenic parasite lines for biological differences.
Blinding	Investigators were blinded to group allocation during data processing.

Reporting for specific materials, systems and methods

We require information from authors about some types of materials, experimental systems and methods used in many studies. Here, indicate whether each material, system or method listed is relevant to your study. If you are not sure if a list item applies to your research, read the appropriate section before selecting a response.

Materials & experimental systems

n/a	Involved in the study
☐	☒ Antibodies
☐	☒ Eukaryotic cell lines
☒	☐ Palaeontology and archaeology
☒	☐ Animals and other organisms
☒	☐ Clinical data
☒	☐ Dual use research of concern
☒	☐ Plants

Methods

n/a	Involved in the study
☒	☐ ChIP-seq
☐	☒ Flow cytometry
☒	☐ MRI-based neuroimaging

Antibodies

Antibodies used	HA epitope tag. Biolegend. Catalog: 901515. Clone: 16B12. Lot: B294011 (https://www.biolegend.com/en-us/products/anti-ha-11-epitope-tag-antibody-11071). anti-mouse HRP secondary antibody (Cytiva NA931-1mL). Dilutions are noted in Methods.
Validation	These antibodies are commercially available and have been extensively validated. We validated this antibody by testing against P. falciparum lysates using western blots as shown in Supplementary Fig. 5b.

Eukaryotic cell lines

Policy information about cell lines and Sex and Gender in Research

Cell line source(s)	Dd2 binding site mutant and Dd2 PfK13 R539T lines were reported by Straimer et al. Science 2015. The NF54_attb2_T7 polymerase_CRISPR/Cas9 parasite line was reported by Polino et al. ACS Infect. Dis. 2020. The PfMnmA conditional knockdown line was generated in this study as described in the Methods.
Authentication	The Pfk13 locus for the Dd2 and Dd2_R539T parasites was tested via Sanger sequencing prior to each experiment to ensure the correct lines. The PfMnmA conditional knockdown line was validated via the PCR strategy in Supplementary Fig. 5a and via Sanger sequencing. Prior to experiments, all four PCR reactions were run to validate the line and ensure the aptamers were intact.
Mycoplasma contamination	All lines tested negative for Mycoplasma contamination.
Commonly misidentified lines (See ICLAC register)	No commonly misidentified lines in this study.

Flow Cytometry

Plots

Confirm that:

☒ The axis labels state the marker and fluorochrome used (e.g. CD4-FITC).

☒ The axis scales are clearly visible. Include numbers along axes only for bottom left plot of group (a 'group' is an analysis of identical markers).

☒ All plots are contour plots with outliers or pseudocolor plots.

☒ A numerical value for number of cells or percentage (with statistics) is provided.

Methodology

Sample preparation	Plasmodium falciparum parasite survival was assessed on an iQue plus flow cytometer (Sartorius) and a BD FACSCelesta flow

Sample preparation	cytometer using SYBR Green I and Mitotracker Deep Red FM (Thermofisher Scientific) as stains for nuclear DNA and mitochondrial activity, respectively.
Instrument	iQue Plus Flow Cytometer (Sartorius) and BD FACSCelesta Flow Cytometer (BD Biosciences)
Software	FlowJo Version 10 (FlowJo LLC)
Cell population abundance	Flow cytometry was used to quantify the percentage of live, fluorescent-labeled parasites in each sample. Parasitemias ranged from 0-10% at varying drug inhibitor concentrations. This method was not used for cell sorting.
Gating strategy	This is a well established gating method routinely used to measure parasitemias, as previously described (Straimer el al. Science, 2015). Flow counts were first gated for red blood cells using FSC and SSC. Live parasites were determined as positive events for BL1-A or FITC-A (SYBR Green) and RL1-A or APC-A (Mitotracker Deep Red) for the iQue or the BD FACSCelesta, respectively. This corresponds to the upper right quadrant of the flow plot. Percentage of parasites was calculated as the number of live parasite events divided by the total red blood cell events for each sample. Supplementary Fig. 12 shows an example of the gating strategy to quantify live parasites.

☒ Tick this box to confirm that a figure exemplifying the gating strategy is provided in the Supplementary Information.
